# Fire-Resistant Coatings: Advances in Flame-Retardant Technologies, Sustainable Approaches, and Industrial Implementation

**DOI:** 10.3390/polym17131814

**Published:** 2025-06-29

**Authors:** Rutu Patel, Mayankkumar L. Chaudhary, Yashkumar N. Patel, Kinal Chaudhari, Ram K. Gupta

**Affiliations:** 1 National Institute for Materials Advancement, Pittsburg State University, 1204 Research Road, Pittsburg, KS 66762, USA; 2 Department of Physics, Pittsburg State University, 1701 S Broadway St, Pittsburg, KS 66762, USA; 3 Department of Chemistry, Pittsburg State University, 1701 S Broadway St, Pittsburg, KS 66762, USA

**Keywords:** fire-resistant, flame-retardant, coating, halogenated, self-healing

## Abstract

Fire-resistant coatings have emerged as crucial materials for reducing fire hazards in various industries, including construction, textiles, electronics, and aerospace. This review provides a comprehensive account of recent advances in fire-resistant coatings, emphasizing environmentally friendly and high-performance systems. Beginning with a classification of traditional halogenated and non-halogenated flame retardants (FRs), this article progresses to cover nitrogen-, phosphorus-, and hybrid-based systems. The synthesis methods, structure–property relationships, and fire suppression mechanisms are critically discussed. A particular focus is placed on bio-based and waterborne formulations that align with green chemistry principles, such as tannic acid (TA), phytic acid (PA), lignin, and deep eutectic solvents (DESs). Furthermore, the integration of nanomaterials and smart functionalities into fire-resistant coatings has demonstrated promising improvements in thermal stability, char formation, and smoke suppression. Applications in real-world contexts, ranging from wood and textiles to electronics and automotive interiors, highlight the commercial relevance of these developments. This review also addresses current challenges such as long-term durability, environmental impacts, and the standardization of performance testing. Ultimately, this article offers a roadmap for developing safer, sustainable, and multifunctional fire-resistant coatings for future materials engineering.

## 1. Introduction

In recent decades, fire safety has become a critical consideration across various industries, including aerospace, automotive, textiles, and construction [1,2,3,4,5]. Polymers, extensively used in these sectors due to their versatility and lightweight nature, inherently suffer from high flammability. During combustion, they can release significant heat and toxic gases, posing severe risks to both human life and property. To address these challenges, integrating FR measures into polymeric systems has emerged as a key safety strategy. Among the various approaches, the application of fire-resistant coatings has proven to be one of the most effective methods for achieving this goal. These coatings act as a surface-level barrier against thermal degradation, ignition, and flame propagation. By focusing protection at the surface, the most vulnerable ignition zone, without compromising the bulk properties of the underlying material, fire-resistant coatings offer a high-performance and material-efficient solution [6]. Historically, halogenated FRs, particularly polybrominated diphenyl ethers (PBDEs), gained popularity due to their high efficacy in disrupting combustion in the gas phase. However, the increasing evidence of their environmental persistence, bioaccumulation, and toxic emissions, such as dioxins released upon burning, has prompted global regulatory restrictions and outright bans in many regions [7]. This shift has spurred intense research into halogen-free alternatives, primarily based on phosphorus, nitrogen, silicon, and other inorganic compounds. These new systems not only offer environmental and health benefits but also present opportunities for multifunctionality, enabling simultaneous improvements in thermal stability, mechanical performance, and environmental compatibility [8]. Fire-resistant coatings are especially valuable because they reduce fire hazards without significantly compromising the weight, flexibility, or esthetics of the host material. Unlike bulk additives, which often require high loadings that can compromise mechanical properties, surface-applied coatings localize the flame resistance at the ignition interface, resulting in improved efficiency and reduced material usage [9]. Additionally, these coatings are compatible with a broad range of substrates, including textiles, foams, metals like steel, wood, and various plastics, which underscores their versatility in diverse industrial applications [10]. Fire-resistant coating systems can generally be categorized into four main types: intumescent, non-intumescent, ultraviolet (UV)-curable, and hybrid technologies. Among these, intumescent coatings are the most widely used due to their dynamic protective mechanism. Upon an exposure to heat, these coatings expand to form a thick, multicellular char layer that thermally insulates the underlying substrate. The formulation of such coatings typically involves an acid source, such as ammonium polyphosphate (APP), a carbon source, such as pentaerythritol (PER), and a blowing agent, like melamine (MA), which together facilitate the intumescence and robust char formation process [11]. In contrast, non-intumescent coatings rely on different mechanisms, such as the release of inert gases or the formation of glass, and protective layers. These systems commonly include mineral fillers, such as aluminum hydroxide (Al(OH)_3_) or magnesium hydroxide (Mg(OH)_2_). These compounds absorb substantial heat during decomposition and release water vapor, which dilutes the concentration of flammable gases and acts as a fire suppressant [12]. UV-curable fire-resistant coatings have also gained attention, particularly in applications that require fast, solvent-free processing, such as electronics, textiles, and architectural elements. These systems often utilize phosphorus-containing monomers and demonstrate excellent adhesion, minimal volatile organic compound (VOC) emissions, and a rapid curing under UV exposure [13]. Another innovative class includes layer-by-layer (LbL) assembled coatings, which offer nanoscale control over the FR architecture. These coatings typically consist of alternating layers of organic and inorganic components, such as chitosan (CS), graphene oxide (GO), and layered double hydroxides (LDHs), that synergistically enhance the flame resistance, barrier properties, and mechanical performance [10]. Understanding the fire hazards associated with different materials is essential for tailoring fire-resistant coatings. Thermoplastics, such as polyethene and polypropylene, are prone to melting and dripping during combustion, which can facilitate flame spread and secondary ignition. On the other hand, thermosets tend to emit high-energy volatile compounds that can intensify fire conditions. Consequently, fire-resistant coatings must be customized according to the specific thermal degradation behaviors of target materials, striking a balance between ignition resistance, stable char formation, and smoke suppression [14]. This review aims to present a comprehensive and up-to-date examination of the recent advances in fire-resistant coatings, focusing on key aspects, including the chemical composition, structural design, underlying mechanisms, and commercial viability. The primary objectives of this review are to present a holistic understanding of fire-resistant coatings by combining scientific fundamentals with practical advancements. To begin with, this review provides a thorough overview of fire chemistry and flame propagation mechanisms as they relate to protective coating systems. This foundation is crucial for understanding the role of various fire-resistant strategies and assessing their relative effectiveness. This review then delves into the core mechanisms of flame retardancy, namely gas-phase inhibition, condensed-phase protection, endothermic cooling, and char formation, which are instrumental in mitigating flammability and suppressing the fire spread. Furthermore, it examines recent innovations in synergistic systems that leverage nanomaterials and bio-based additives to enhance flame retardancy through multifunctional and sustainable approaches. In light of growing environmental and regulatory concerns, this review places a particular emphasis on eco-friendly FRs, evaluating their potential for industrial scalability and compliance with evolving safety standards. Finally, application-specific coating systems are discussed in terms of the fire hazards they aim to mitigate, as well as their performance requirements, thereby bridging the gap between scientific research and real-world implementation. Through these objectives, this review serves as a valuable resource for materials scientists, chemical engineers, fire protection specialists, and policymakers alike. Moreover, by consolidating recent research developments and aligning them with practical fire safety requirements, this review aims to guide the development of next-generation fire-resistant coatings that are not only effective and scalable but also environmentally and economically sustainable.

## 2. Fire-Resistant Polymers

### 2.1. Fundamentals of Flame Retardancy

The combustion of polymers is a multifaceted process that unfolds in several stages, including thermal degradation (pyrolysis), vapor-phase ignition, flame propagation, and the heat feedback to the substrate. Initially, the application of heat leads to the breakdown of polymer chains into VOCs. These volatiles mix with atmospheric oxygen and combust, releasing significant amounts of heat, smoke, and toxic gases [15]. The fire tetrahedron model provides a framework for understanding this process by identifying the four essential components required for sustaining a fire: fuel, oxygen, heat, and free radicals. Among these, free radicals such as hydrogen (H•) and hydroxyl (OH•) play a pivotal role in accelerating the combustion chain reactions. At the same time, the heat generated is fed back into the system to perpetuate further pyrolysis. FRs exert their protective effect by disrupting one or more of these elements through mechanisms like radical scavenging, cooling, or the formation of protective barriers [6]. Gas-phase inhibition is one of the primary strategies employed by FRs to disrupt the radical chain reactions responsible for sustaining flames. Phosphorus-containing compounds are particularly effective in this regard; upon thermal decomposition, they produce PO• radicals that capture and neutralize the highly reactive H• and OH• radicals, thus reducing the flame propagation and intensity. While halogenated FRs have historically been effective in inhibiting gas-phase fires, they are increasingly being phased out due to concerns over environmental persistence and toxicity [16]. In the condensed phase, the FR action typically involves the formation of a stable char layer on the surface of the substrate. This char acts as a thermal and physical barrier, restricting both the heat and mass transfer and thereby limiting the availability of fuel and oxygen required to sustain combustion. Additives such as APP and PER are widely used in intumescent systems to facilitate efficient char formation [17]. Another important mechanism is endothermic dilution, achieved using additives like Mg(OH)_2_ and Al(OH)_3_. These substances undergo endothermic decomposition, absorbing heat from the burning substrate and releasing water vapor. The released vapor cools the burning surface and dilutes the local concentration of flammable volatiles, thus retarding the combustion process [18]. Char formation and intumescence often act in synergy to provide enhanced fire protection. Intumescent coatings, which typically incorporate a tripartite formulation of APP, PER, and MA, rely on a coordinated sequence of actions: the acid-catalyzed dehydration of the carbon source, blowing agent-induced foaming, and the stabilization of the resulting char layer. This sequence results in a swollen, foamed char that effectively isolates the substrate from flames and heat [11]. The inclusion of synergistic additives further amplifies the effectiveness of FR systems. For example, nano-LDHs used in conjunction with APP/PER-based intumescent formulations can significantly enhance char cohesion and thermal stability [18]. Additionally, nanomaterials such as GO, silica (SiO_2_) nanoparticles, and iron oxides have been shown to promote a better dispersion of active ingredients, increase barrier properties, and catalyze the formation of a more robust char layer [19]. Among the various classes of halogen-free FR systems, intumescent coatings remain the most prominent and effective. Their functionality is derived from a complex yet well-coordinated mechanism involving acid catalysis, thermal expansion through blowing agents, and char layer stabilization. The performance of these coatings can be further improved by using catalysts such as titanium dioxide (TiO_2_) and iron oxide, which contribute to the compactness of the char residue and its barrier effectiveness [20]. Overall, a thorough understanding of these fundamental FR mechanisms, including gas-phase inhibition, condensed-phase protection, endothermic dilution, char formation, and synergistic enhancement, provides critical insights for the design and development of next-generation coatings. These advanced systems are expected to offer not only superior fire protection but also environmental sustainability and industrial scalability, aligning with current regulatory and performance standards.

### 2.2. Classification of Flame Retardants

FRs in coatings can be classified into several categories based on their chemical composition, mechanism of action, and functionality within the coating matrix. As polymer-based materials continue to dominate in construction, electronics, textiles, and transportation, the demand for highly efficient, environmentally benign fire-resistant coatings has surged. Modern coatings integrate FRs not only to delay ignition but also to suppress smoke generation, inhibit the flame spread, and reduce the release of toxic gases during combustion. The effectiveness of these FRs depends heavily on their structural chemistry, thermal degradation behavior, and interaction with polymer matrices. From traditional halogenated compounds to advanced synergistic systems, FRs have evolved to meet stringent fire safety regulations and environmental expectations [21].

#### 2.2.1. Halogenated Flame Retardants

**Figure 1:**
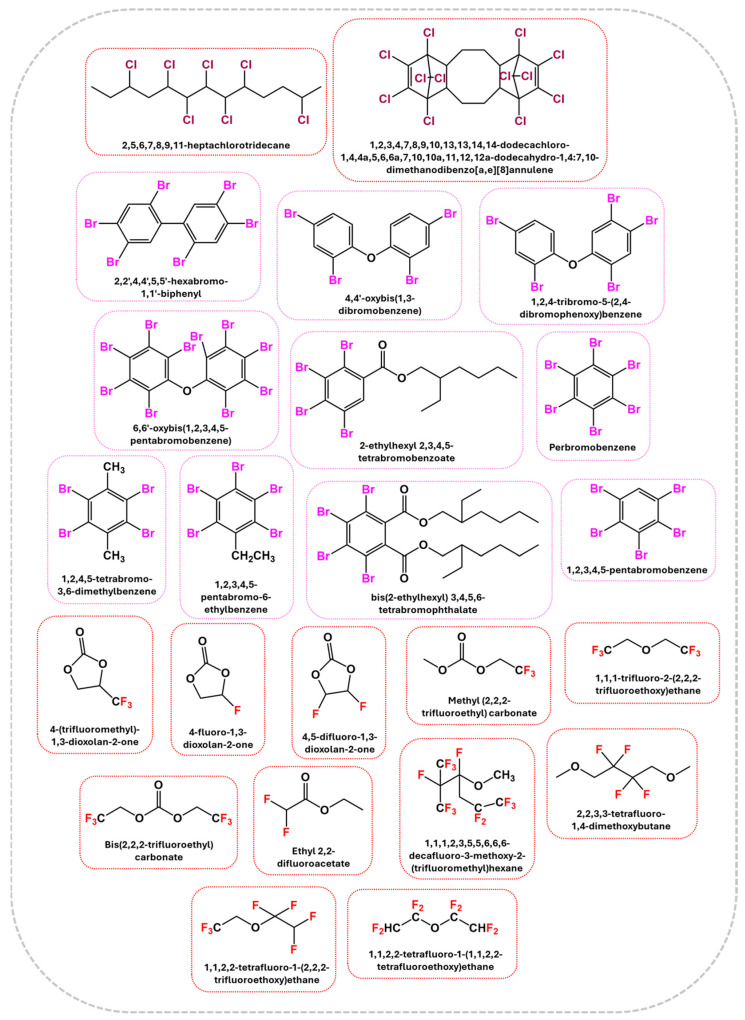
Halogen-based FRs.

Halogenated flame retardants (HFRs) are a class of chemicals that contain bromine or chlorine, commonly incorporated into materials to inhibit or delay combustion. Since the 1970s, these additives, particularly brominated flame retardants (BFRs), have been extensively used to comply with fire safety standards such as California’s Technical Bulletin 117. HFRs have been integrated into consumer products, such as polyurethane (PU) foam used in furniture and commercial textiles, to mitigate fire risks. While BFRs have played a crucial role in fire prevention and life safety, growing concerns regarding their environmental persistence and potential health hazards have sparked scrutiny about their widespread use and long-term effects [22]. Figure 1 shows chemical structures of halogen-based FRs [22,23,24,25].

HFRs are valued for their gas-phase FR action, cost-effectiveness, and high efficiency and were among the first additives used in fire-resistant PU systems [26]. During combustion, PU releases highly reactive free radicals such as •OH and •H that propagate the flame. HFRs thermally decompose to produce hydrogen halides (hydrogen bromide (HBr) and hydrogen chloride (HCl)), which react with these radicals to form less reactive species, interrupting the chain reaction and extinguishing the flame. Brominated compounds further enhance the flame retardancy in the condensed phase by promoting char formation, which acts as a physical barrier against heat and oxygen diffusion. BFRs, such as tetrabromobisphenol A (TBBPA) and hexabromocyclododecanes (HBCDs), are the most used due to their superior radical-trapping efficiency at relatively low decomposition temperatures. TBBPA undergoes complex degradation pathways involving isopropylidene bond cleavage and C–Br bond scission, releasing brominated phenols and HBr while generating a protective carbonaceous char layer. The release of HBr enables effective gas-phase inhibition by terminating chain-propagating radicals, and kinetic studies have shown that these reactions can significantly slow down flame propagation. HBCDs follow a similar pathway, degrading through autocatalytic radical processes that lead to the release of multiple brominated cyclic species and char. Despite their effectiveness, TBBPA and HBCDs are under regulatory scrutiny due to their potential for bioaccumulation and environmental persistence. This has led to the development of next-generation BFRs with a lower toxicity, such as decabromodiphenyl ethane and bis(2-ethylhexyl)tetrabromophthalate. Although chlorinated flame retardants (CFRs) are generally less efficient than BFRs, they still contribute significantly to PU flame retardancy. Their mechanism involves the thermal degradation to HCl, which similarly reacts with flame radicals. Compounds such as chlorinated paraffins (CPs), chlorendic anhydride, and tris(2-chloroethyl) phosphate are common CFRs used as either additives or reactive FRs. CPs undergo dehydrochlorination during combustion, releasing HCl and forming conjugated double bonds that facilitate the creation of crosslinked char. However, the stronger C–Cl bonds require higher activation energy for cleavage, limiting their efficiency in the gas phase compared to BFRs. Additionally, the environmental persistence and potential carcinogenicity of certain CFRs, particularly short-chain CPs and chlorinated organophosphates, have prompted regulatory restrictions and a growing shift toward safer alternatives. In conclusion, while halogenated FRs remain highly effective for enhancing fire resistance in PU materials, their associated environmental and health risks necessitate responsible use and continuous innovation. The ongoing research focuses on designing halogenated compounds with a reduced migration potential and toxicity, as well as utilizing synergistic agents such as metal oxides or phosphorus-containing additives to enhance performance further. Nonetheless, due to their potent flame-inhibiting action and low required concentrations, HFRs continue to serve as critical components in commercial fire safety systems, particularly in high-risk applications requiring stringent regulatory compliance [27].

#### 2.2.2. Inorganic Additives as Flame Retardants

**Figure 2:**
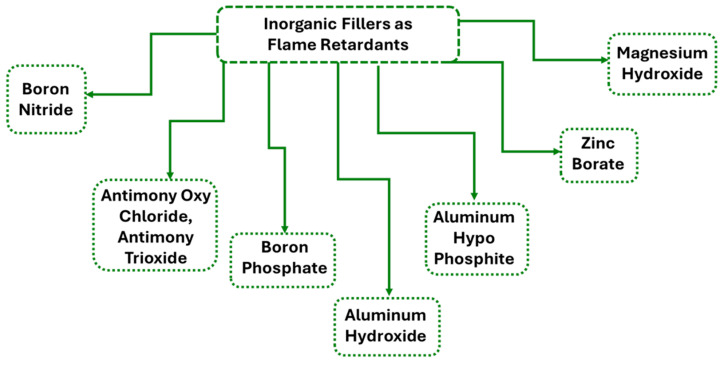
Inorganic fillers as FRs.

Inorganic metal hydroxides, particularly Al(OH)_3_ and Mg(OH)_2_, are among the most widely used FRs in plastics, cables, construction materials, and transportation due to their non-toxic nature and cost-effectiveness. These materials function by undergoing endothermic decomposition upon heating, releasing water vapor that dilutes flammable gases and cools the material. Simultaneously, the formation of residual metal oxides (Al_2_O_3_ and MgO) contributes to a protective barrier that inhibits flame spread and lowers the surface temperature of the polymer. Al(OH)_3_ decomposes at 190–230 °C, making it suitable for polymers processed at temperatures below this range. In contrast, Mg(OH)_2_ possesses a higher decomposition temperature (~300 °C), making it more appropriate for high-temperature applications, including thermoplastics and thermosets. Although these hydroxides effectively reduce the heat release and toxic fume generation, they generally require high loading levels and processing at relatively low temperatures (around 120 °C) to achieve an optimal performance [28]. A recent study by Scionti et al. demonstrated that surface-modified magnesium hydroxide (Mg(OH)_2_-S) outperformed other fillers in acrylic-based fire-resistant coatings. Mg(OH)_2_-S exhibited the lowest backside temperature during flame exposure and produced the smallest fire-damaged area among all tested materials. This enhanced performance is attributed to Mg(OH)_2_-S’s higher heat absorption during decomposition (1389 J/g compared to 1050 J/g for Al(OH)_3_) and its higher decomposition onset (~332 °C), which aligns more closely with typical fire conditions. Consequently, Mg(OH)_2_-S acts as a more effective thermal barrier, prolonging the structural integrity of the coating under direct flame exposure. These properties make Mg(OH)_2_-S a particularly attractive FR for demanding applications, such as naval or industrial coatings, where high fire resistance and thermal insulation are critical [29]. Figure 2 displays examples of inorganic fillers that can be used as FRs [30,31,32,33,34,35,36].

#### 2.2.3. Phosphorus-Based Systems

**Figure 3:**
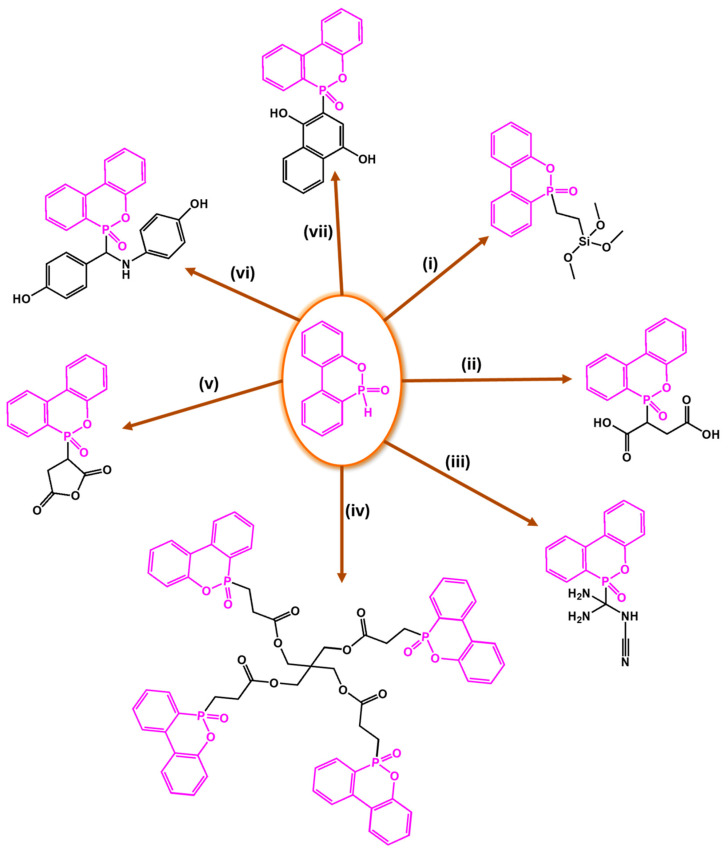
Some derivatives of DOPO (1), synthesized via Michael-type additions: (i) (aVinyltri methoxysilane (benzene, 80 °C) and Azobisisobutyronitrile (benzene, 80 °C; 12 h). (ii) Maleic acid (xylene: tetrahydrofuran (THF) (1:1), 80 °C; 20 h). (iii) Dicyandiamide (DICY) (130 °C). (iv) Toluene (s) (100 °C); Tetra [(acryloyl-oxy)ethyl] pentaerythrit (toluene, 100 °C; 2 h); and 200 °C (10 mbar; 6 h). (v) Maleic anhydride (THF; reflux) or using xylene (80 °C; 24 h). (vi) 4-aminophenol, 4 hydroxybenzaldehyde (methanol, 50 °C; 5 h), the product from was then used for the next step (THF, 60 °C; 12 h). (vii) 1,4-naphthoquinone (inert solvent, dielectric constant ≤10) [37]. Copyright 2014 Elsevier.

**Figure 4:**
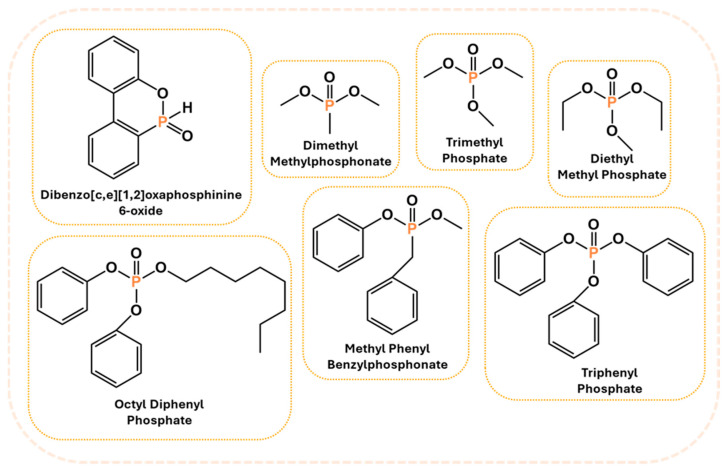
Phosphorus-based FRs.

Phosphorus-containing FRs have emerged as environmentally friendly alternatives to toxic halogenated systems. Among them, organophosphorus compounds based on 9,10-dihydro-9-oxa-10-phosphaphenanthrene-10-oxide (DOPO) have been extensively studied due to their high FR efficiency and thermal stability. Numerous synthetic routes have yielded a variety of DOPO derivatives (Figure 3), offering tailored structures for specific polymer applications. These phosphorus-based FRs often benefit from synergistic effects when combined with nitrogen-containing compounds, further enhancing the flame inhibition. One key advantage of non-halogenated phosphorus systems, such as DOPO derivatives, is their reduced environmental and toxicological impact. During combustion, phosphorus is retained within the char matrix, thereby limiting the formation of toxic gases and smoke [37]. Aromatic phosphonate FRs derived from DOPO exhibit excellent thermal stability, with decomposition temperatures typically exceeding 250 °C. This makes them suitable for use in high-temperature processable polymers, such as polyester and polyamides. Moreover, DOPO-based compounds often incorporate P–C bonds, contributing to their structural rigidity and thermal endurance. Although a broad array of phosphorus-containing FRs have been developed, further research is necessary to address key challenges that hinder large-scale commercialization. These include the need for cost-effective synthetic strategies, the comprehensive evaluation of mechanical properties, compatibility with polymer matrices, long-term durability, and a thorough understanding of their toxicological and environmental profiles [38]. Figure 4 shows phosphorus-based FRs [25,39].

#### 2.2.4. Nitrogen-Based Systems

**Figure 5:**
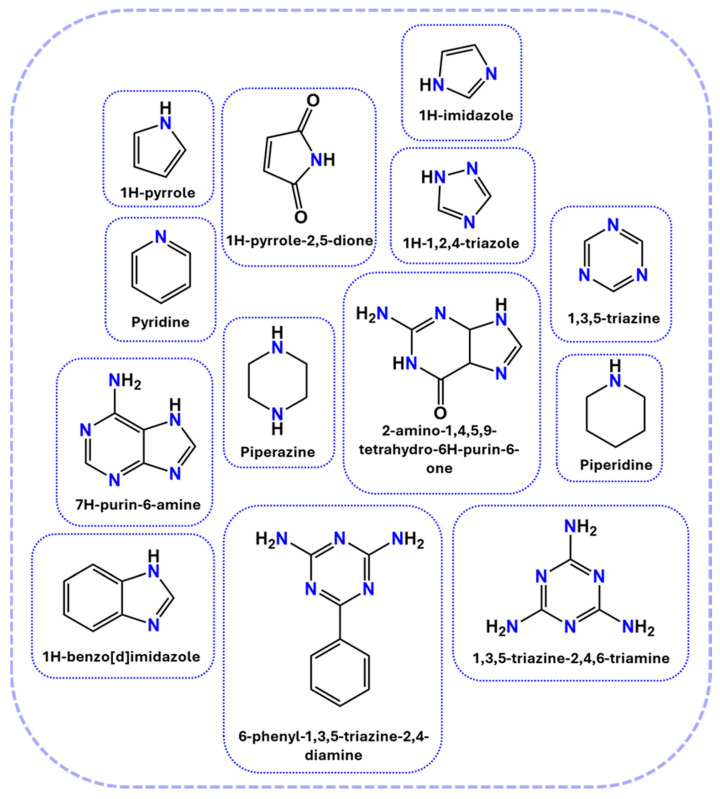
Nitrogen-based FRs.

Nitrogen-based FRs are known for their ability to release inert gases during decomposition, thereby diluting flammable volatiles and suppressing combustion in the gas phase. Among these, MA and DICY are widely studied due to their high nitrogen content and char-forming capabilities. The examples of nitrogen-based FRs are illustrated in Figure 5 [40,41,42].

In a study by Xu et al., a novel ternary FR system was developed by coating zeolitic imidazolate framework-8 (ZIF-8) with MA (forming ZIF-8@MA) and subsequently bonding it with diatomite to generate a Si–N–Zn composite structure. The resulting material (ZMD) was incorporated into a rigid PU foam, significantly enhancing its fire safety. MA contributed by releasing non-combustible gases during thermal degradation, which helped lower the combustion temperature and dilute flammable gases. The synergistic effect of ZMD was evident in the reduction in critical fire parameters: the peak heat release rate (PHRR), total heat release (THR), smoke production rate (SPR), and total smoke production (TSP) were reduced by 50.1%, 61.8%, 70.6%, and 76.1%, respectively. Furthermore, the limiting oxygen index (LOI) increased from 19.4% to 25.4%. These improvements were attributed to the gas-phase action of MA, the catalytic char formation by zinc oxide (ZnO) and SiO_2_, and the physical barrier effect of diatomite [43]. DICY is another nitrogen-rich FR that enhances fire resistance by releasing inert gases such as ammonia and nitrogen during decomposition while simultaneously promoting carbonaceous char formation. When combined with aluminum hypophosphite (AHP) in a 4:1 weight ratio within polyethene composites, DICY demonstrated excellent flame retardancy. The LOI increased from 18.8% to 26.3%, a UL-94 V-0 rating was achieved, the PHRR dropped by 48%, and the char residue increased to 23.2%. These results indicate a strong synergistic effect between DICY and AHP, improving both the thermal stability and flame suppression efficiency [44].

In a broader context, Gilbertson and Ng evaluated alternatives to traditional BFRs, such as decabromodiphenyl ether and TBBPA, in electrical and electronic equipment using a comprehensive alternatives assessment framework. This study compared non-halogenated candidates, including organophosphorus compounds (e.g., DOPO and MA polyphosphate), mineral fillers (e.g., montmorillonite (MMT)), and nanomaterials (e.g., carbon nanotubes (CNTs)). TBBPA was identified as the most hazardous among the evaluated substances. While DOPO and melamine polyphosphate (MPP) exhibited a lower toxicity, the absence of complete hazard and exposure data constrained their evaluation. Nanomaterials demonstrated a promising performance but raised concerns regarding long-term safety and regulatory gaps. The authors emphasized the importance of integrating green chemistry principles into future assessment frameworks to guide the development of safer, sustainable FR alternatives [45].

#### 2.2.5. Silicone-Based Flame Retardants

**Figure 6:**
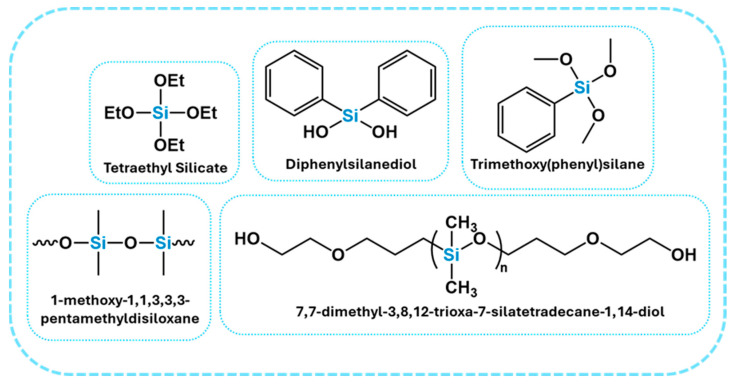
Silicone-based FRs.

Silicone-based FRs are an emerging class of environmentally friendly additives that provide an excellent thermal stability, char formation, and surface protection. Their FR mechanism involves the creation of thermally stable silicon-containing residues, such as SiO_2_ or Si–O/Si–N ceramics, which function as insulating layers, reducing the heat and oxygen transfer during combustion. These systems are non-halogenated and often multifunctional, contributing not only to flame resistance but also to the hydrophobicity, weatherability, and mechanical enhancement of the coating or polymer system. Figure 6 represents examples of silicone-based FRs [46,47,48,49,50].

**Figure 7:**
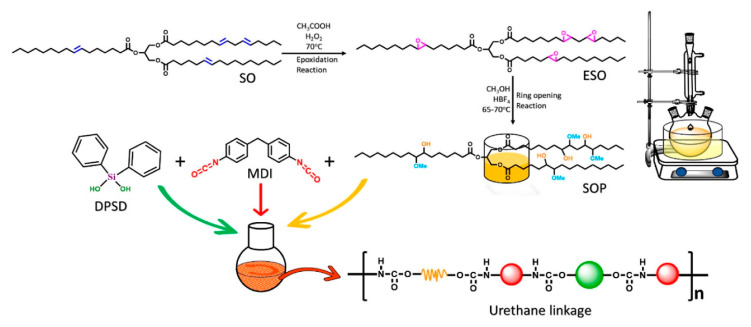
The reaction scheme of the PU coating material [47]. Copyright 2024 American Chemical Society.

In a study by Patel et al., diphenylsilanediol (DPSD) served as a reactive FR in biobased PU coatings. Rich in silicon, DPSD enhances flame retardancy by promoting the formation of a SiO_2_-like char barrier upon thermal exposure, which slows the heat and mass transfer during combustion. The incorporation of DPSD significantly delayed the ignition time, reduced the burning duration, and minimized weight loss, indicating an improved thermal stability. Its two hydroxyl groups facilitated covalent bonding with isocyanate groups in the PU matrix, resulting in a highly crosslinked network with an enhanced mechanical hardness, solvent resistance, and durability. Furthermore, the presence of hydrophobic phenyl groups and a siloxane backbone imparted low surface energy, creating a coating with hydrophobic and anti-smudge properties, including a high water contact angle and easy ink removal. Importantly, DPSD is fluorine-free and compatible with renewable feedstocks, such as soybean oil-based polyols, making it a sustainable alternative to conventional HFRs. Figure 7 shows the reaction scheme of the PU coating with DPSD [47].

In another study, Chatterjee et al. investigated the use of polysilazane (PSZ) as a silicon-based FR and curing agent in wood acrylic coatings. PSZ features a Si–N backbone that transforms into ceramic-like Si–O or Si–N structures upon thermal degradation, creating effective barriers to heat and oxygen. When used in combination with 3 wt% SiO_2_, PSZ imparted a synergistic effect that raised the LOI to 26% and achieved a UL-94 V-0 rating. Furthermore, PSZ enhanced the thermal stability, char formation, and hydrophobicity of the coating while maintaining mechanical robustness and long-term weather resistance. Notably, the system avoided toxic isocyanate crosslinkers, aligning with the principles of green chemistry and sustainable materials design [51].

### 2.3. Hybrid and Synergistic Flame Retardants

Hybrid and synergistic FR systems combine two or more additives to achieve superior flame retardancy through complementary mechanisms. These systems often enhance the thermal stability, char formation, and mechanical performance while reducing additive loading and preserving material properties.

**Figure 8:**
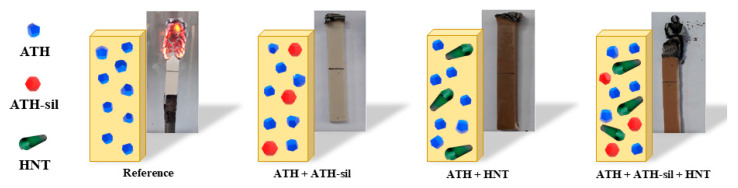
Hybrid FR system based on HNTs and silane-modified ATH [52]. Copyright 2021 MDPI. Licensed under CC BY. https://creativecommons.org/licenses/by/4.0/ (accessed on 28 June 2025).

In a study by Paszkiewicz et al., a hybrid FR system comprising halloysite nanotubes (HNTs) and silane-treated alumina trihydrate (ATH-sil) was developed for ethylene–vinyl acetate/copolymer/low-density polyethylene composites used in cable insulation. Both additives are halogen-free and environmentally friendly. The optimal formulation (8 wt% HNT + 4 wt% ATH-sil) significantly increased the LOI to over 28%, achieving self-extinguishing behavior. It also raised the 50% degradation temperature from 467 °C to 520 °C. ATH-sil improved the filler dispersion and interfacial bonding, while HNTs acted as thermal barriers. Together, they contributed to an enhanced mechanical strength, reduced water absorption, and improved processability (Figure 8). The hybrid FR system is based on HNTs and ATH-sil [52]. Furthermore, the EVA/AGM composite comprising Al(OH)_3_, molybdenum disulphide (MoS_2_), and graphene nanoplatelets (GNPs) was identified as the most effective FR system in the same study. This formulation (36 wt% Al(OH)_3_, 2 wt% MoS_2_, and 2 wt% GNPs) achieved a UL-94 V-0 rating with the lowest Al(OH)_3_ loading reported. The superior performance was attributed to the synergistic action of MoS_2_ and GNPs: GNPs promoted char formation and acted as physical barriers, while MoS_2_ enhanced the thermal stability and facilitated the formation of a protective layer. This synergy significantly reduced the heat release and improved both thermal and mechanical properties, making EVA/AGM a promising candidate for high-performance fire-resistant cable sheathing [53]. Despite the promising FR performance and mechanical enhancement offered by hybrid systems, several challenges remain that can limit their practical implementation. One key issue is the interfacial compatibility between inorganic fillers and the polymer matrix, which can lead to poor dispersion or weak filler–matrix interactions, ultimately affecting the long-term mechanical integrity of the composite. For instance, achieving the uniform dispersion of nanofillers like MoS_2_ or GNPs requires surface modifications or compatibilizers, which may introduce additional processing steps. Moreover, processing challenges such as an increased melt viscosity, filler agglomeration, and poor extrusion flow behavior can hinder scalability in industrial settings. The presence of multiple fillers can also complicate the optimization of processing parameters like the mixing time, temperature, and shear rates. Therefore, while hybrid and synergistic FR systems offer clear benefits, addressing these material compatibilities and processability issues remains critical for their widespread application in commercial polymer systems [54,55,56,57]. Table 1 displays some literature data from previously reported work on FRs.

## 3. Coating Matrix Systems and Compatibility

### 3.1. Epoxy-Based Coatings

Epoxy resin is a highly versatile thermosetting polymer that forms a robust three-dimensional network through inter- and intramolecular crosslinking. Due to its adaptable chemistry and processability, epoxy is used across a wide range of applications, including adhesives, protective and decorative coatings, and surface treatments for materials such as wood, fabric, glass, and metal. Its widespread use in household, structural, electronic, and construction products often necessitates compliance with stringent flame safety standards [94]. Epoxy-based PU coatings, which integrate the mechanical strength of epoxy with the flexibility and UV resistance of PU, provide durable and protective finishes for various substrates.

Laoutid et al. investigated the use of epoxy resin as a matrix for transparent fire-resistant coatings by incorporating hydroxyapatite nanoparticles. The epoxy system was based on the diglycidyl ether of bisphenol A (DGEBA, EPIKOTE Resin 828), cured with EPIKURE F205. This thermoset exhibited an excellent chemical resistance, strong substrate adhesion to PLA, and effective dispersion of nanofillers. AHP nanoparticles (≤60 nm) were incorporated through a two-step wet milling process, followed by sonication and mechanical stirring. At an optimal loading of 15 wt% AHP, the resulting epoxy–AHP nanocomposite significantly reduced the PHRR of PLA while maintaining an optical transparency. The matrix not only served as a binder but also aided the char formation during decomposition, emphasizing the importance of additive compatibility in epoxy FR systems [95].

**Figure 9:**
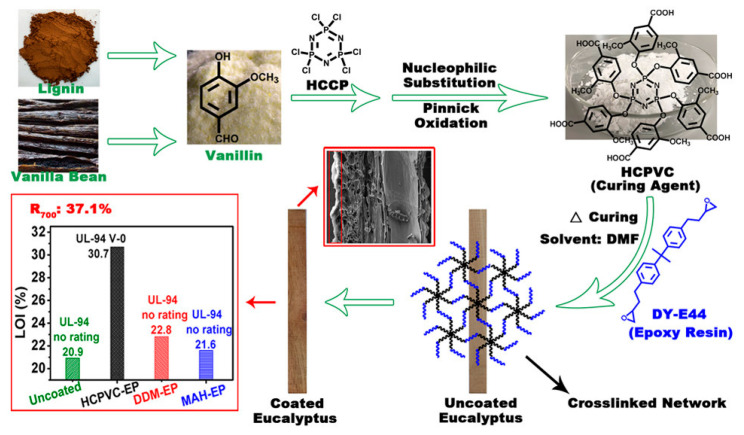
Synthesis of bio-based FR for wood surface coating [96]. Copyright 2019 American Chemical Society.

In another study, Price et al. utilized Epon 828 epoxy resin as a FR matrix due to its excellent thermal stability and char-forming ability. The formulation incorporated intumescent additives, including APP, MA, and PAA. The epoxy matrix successfully confined these components, resulting in thermally stable, expanding coatings. Notably, this method provided a boric acid-free alternative with a superior char integrity and reduced flammability, functioning through an effective condensed-phase FR mechanism [60]. Guo et al. developed a bio-based epoxy fire-resistant coating using DY-E44 epoxy resin, cured with hexa-(4-carboxyl-2-methoxy-phenoxy)-cyclotriphosphazene (HCPVC) and synthesized from vanillin and hexachlorocyclotriphosphazene -. HCPVC acted both as a curing agent and an FR, displaying an excellent compatibility with the epoxy matrix. Differential scanning calorimetry revealed a high activation energy of 71.59 kJ/mol, indicating good reactivity. The cured coatings demonstrated a strong mechanical performance, including a 4H hardness, 5B adhesion, and resistance to water and solvents. A scanning electron microscopy (SEM) analysis confirmed the formation of compact char layers after combustion. The system cured efficiently between 80 and 120 °C and provided a superior flame retardancy, as well as comparable or better physical properties than conventional agents, such as diamino diphenylmethane and maleic anhydride. These results position HCPVC-based epoxy coatings as a sustainable, multifunctional solution for eco-friendly wood protection. The reaction scheme of the biobased epoxy coating using HCPVC is presented in Figure 9 [96].

### 3.2. Polyurethane and Polyurea Coatings

PU and polyurea coatings have emerged as prominent materials in protective applications due to their exceptional mechanical strength, chemical resistance, and environmental durability. These thermosetting systems can be engineered for flexibility or rigidity and are commonly utilized in industrial flooring, automotive parts, and construction. Incorporating FR additives or reactive FR monomers into PU and polyurea matrices facilitates the development of multifunctional coatings that provide both thermal protection and fire resistance, thereby expanding their utility in fire-critical applications.

**Figure 10:**
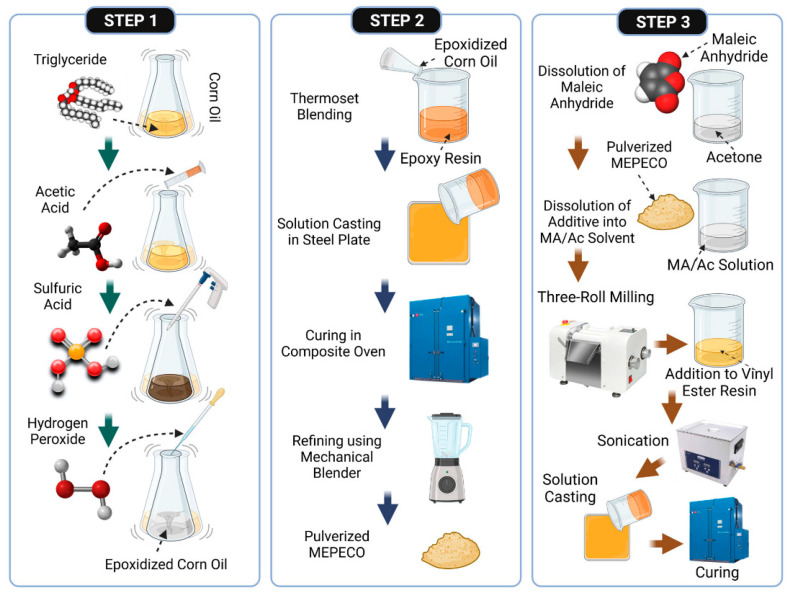
The schematic of the experimental procedures of the synthesis of vinyl ester resins through MEPECO additives [97]. Copyright 2024 American Chemical Society, licensed under CC-BY 4.0. https://creativecommons.org/licenses/by/4.0/ (accessed on 25 June 2025).

In a study by Cabo et al., a bio-based additive system using a maleated epoxidized corn oil/epoxy resin (MEPECO) was introduced into a vinyl ester (VE) resin matrix to enhance its mechanical and FR properties. The VE matrix, a thermoset derived from a blend of an epoxy and unsaturated polyester, was modified with 0–7 wt% in situ-synthesized MEPECO (Figure 10). The effective dispersion via 3-roll milling and enhanced interfacial bonding, facilitated by maleic anhydride groups, resulted in strong chemical crosslinking between MEPECO and the VE network. At an optimal 5 wt% loading, MEPECO improved the thermal stability, reduced the HRR by 17.07%, and enhanced the mechanical integrity. Fourier transform infrared spectroscopy confirmed successful chemical bonding and an adequate defect coverage, validating MEPECO as a multifunctional and sustainable additive [97].

Ding et al. developed a reactive fire-resistant PU foam by chemically integrating a phosphorus–nitrogen-containing molecule, 2-((bis(2-hydroxyethyl)amino)methyl)-5,5-dimethyl-1,3,2-dioxaphosphinane 2-oxide (HAMPP), into the PU matrix. The dihydroxy-functionalized HAMPP reacted covalently with isocyanate groups during the polymer formation, ensuring an excellent compatibility without phase separation or migration. The system demonstrated significant improvements in flame retardancy, with the LOI increasing to 23.7%, the heat release capacity decreasing by 31%, and char production rising by 42%. Furthermore, incorporating 10 wt% HAMPP enhanced the thermal stability and mechanical performance, affirming its potential as a green, matrix-integrated FR for flexible PU foams [98].

### 3.3. Acrylic- and Silicone-Based Coatings

Acrylic- and silicone-based coatings have garnered increasing attention as advanced fire protection systems due to their intrinsic thermal stability, mechanical robustness, weather resistance, and formulation versatility. Acrylics provide excellent adhesion and transparency, while silicones offer heat and UV resistance. The incorporation of functional additives and hybrid organic–inorganic architectures further enhances their FR properties. These coatings are now being customized for sustainable, halogen-free formulations with a low toxicity, minimal smoke release, and superior char formation.

A recent study by Chatterjee et al. introduced a high-performance fire-resistant acrylic coating using PSZ as a crosslinker and SiO_2_ nanoparticles as reinforcing fillers. PSZ exhibited an excellent compatibility with hydroxy-functionalized acrylic resins, forming robust Si–O–C bonds that facilitated efficient ambient-temperature curing and an enhanced coating strength. The inclusion of SiO_2_ provided a synergistic enhancement, significantly increasing the flame retardancy, hydrophobicity, and char formation. The optimized formulation (acryl-PSZ-20) achieved a UL-94 V-0 rating and an LOI of 26%, alongside excellent durability and environmental resistance. This isocyanate-free, eco-friendly system demonstrates substantial potential in wood protection and sustainable FR technologies [99].

**Figure 11:**
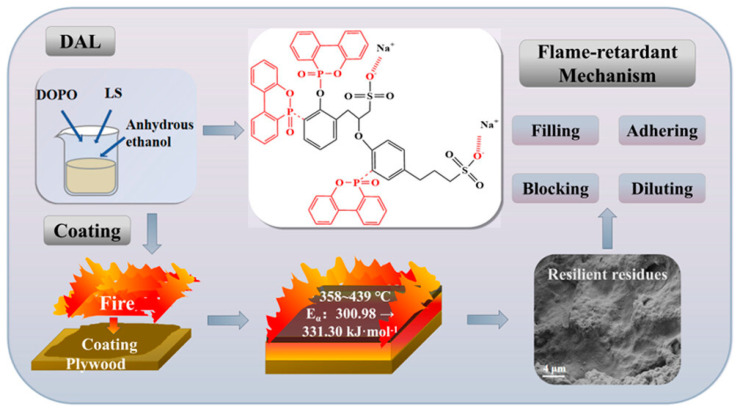
Preparation of phosphorus-modified sodium lignosulfonate intermediates and their integration into Si–P–C silicone–acrylic emulsion coatings for fire-resistant plywood [100]. Copyright 2024 American Chemical Society.

Lai et al. developed a novel FR system based on a silicone–acrylic emulsion (SAE) matrix incorporating two innovative additives: DOPO-modified sodium lignosulfonate (DAL) and carboxymethyl CS-pretreated MPP (CMCS@MPP) (Figure 11). The SAE matrix provided a stable hybrid organic–inorganic network with excellent adhesion, thermal resistance, and weatherability. Hydrogen bonding and ion exchange mechanisms between the additives and matrix enhanced the interfacial compatibility, leading to uniform dispersion and an improved mechanical performance. At an optimal DAL loading of 1.5 wt%, the composite coating demonstrated strong flame retardancy, enhanced adhesion, and the formation of a protective Si–P–C char layer during combustion [100].

In another study, Wang et al. engineered an intumescent fire-resistant coating using a waterborne acrylic matrix integrated with sodium silicate hydrate and melamine cyanurate (MCA). While sodium silicate provides thermal stability and environmental safety, it is often plagued by its poor dispersion and crystallization in organic matrices. The addition of MCA, a nitrogen-rich FR, addressed these challenges by forming hydrogen bonds with sodium silicate. This interaction inhibited sodium silicate polymerization and aggregation, thus enhancing the dispersion and matrix compatibility. The resultant composite coating exhibited an improved flame resistance and thermal stability and maintained its mechanical integrity, demonstrating the advantages of synergistic modifiers in waterborne acrylic systems [101].

### 3.4. Waterborne and Solvent-Based Systems

Waterborne and solvent-based fire-resistant coating systems embody two significant technological approaches, each with unique benefits and constraints. Waterborne coatings, which utilize water as the main carrier, offer reduced VOC emissions, a lower toxicity, and greater environmental safety. However, they may display slower drying times and slightly lower durability. In contrast, solvent-based systems deliver superior adhesion, chemical resistance, and quick curing; however, their reliance on organic solvents leads to higher VOC emissions and potential fire and health risks. Specific performance requirements, environmental regulations, and substrate compatibility typically guide the selection between the two systems.

**Figure 12:**
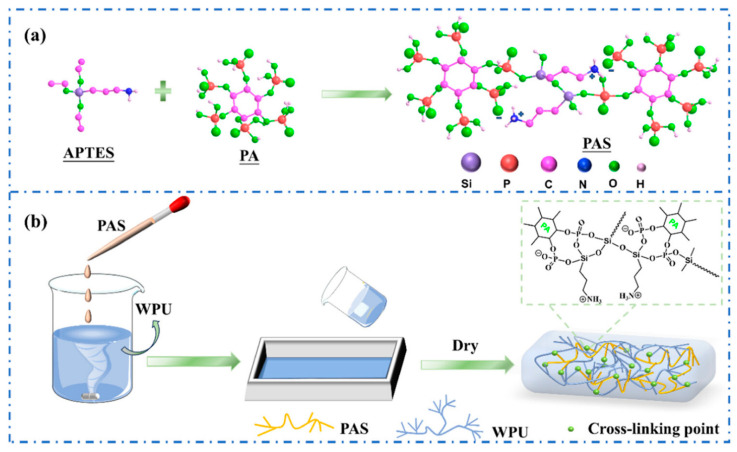
The schematic of the preparation of the (**a**) PAS and (**b**) blended film [102]. Copyright 2025 American Chemical Society.

Yin et al. developed a high-performance waterborne PU (WPU) coating system by incorporating PA and APTES into a phosphorus-rich PU matrix (Figure 12). This bio-based and eco-friendly formulation created a densely crosslinked network through hydrogen bonding and covalent interactions among PA, APTES, and the WPU backbone. The resulting PA-APTES solution (PAS)-WPU coating demonstrated a remarkable FR performance, achieving an LOI of 34.1%, suppressing melt dripping, and enhancing mechanical integrity. Furthermore, the coating proved to be effective on polyester textiles, bolstering both the fire resistance and tensile strength. This study highlights the viability of waterborne systems for sustainable fire-resistant coatings with excellent multifunctionality [102].

Wang et al. synthesized a nanocomposite coating based on lignin-containing waterborne PU by utilizing low-molecular-weight lignin (LMWL) as a bio-based polyol, combined with dialyzed alkali lignin (DAL) nanoparticles. The strong interfacial hydrogen bonding between lignin and PU segments enabled an excellent dispersion and avoided phase separation. LMWL served as a reactive compatibilizer, chemically integrating into the polymer backbone and enhancing the overall matrix homogeneity. This structure significantly improved the thermal stability, photothermal conversion efficiency, and char production. While the primary aim was photothermal energy generation, the resulting system demonstrated a promising FR potential, particularly in environmentally friendly and bio-derived coatings [103].

In another study, Guidugli et al. introduced a solvent-based FR approach by utilizing DESs composed of PA (as the hydrogen bond donor) and various hydrogen bond acceptors, such as choline chloride, ethylene glycol, and glycerol. These DESs were directly applied to cotton fabrics through a straightforward dip-coating process, serving both as the solvent medium and FR agents. Among the tested systems, the ChCl: PA (1:5) composition demonstrated superior fire resistance, low smoke production, and high char formation. The TGA confirmed a high thermal stability, while virtual models for the property evaluation of chemicals within a global architecture-based environmental and toxicological assessments indicated a low toxicity. This solvent-only strategy offers a sustainable and efficient alternative for the FR treatment of cellulose-based materials [104].

### 3.5. Nanocomposite for Flame Retardancy

Nanocomposite-based FR systems have emerged as a potent strategy to enhance the thermal stability, fire resistance, and multifunctionality of polymer coatings. By integrating nanostructured additives, such as clays, metal oxides, graphene derivatives, or hybrid organic–inorganic fillers, into polymer matrices, these systems benefit from an increased surface area, improved dispersion, and synergistic interactions that enhance both the barrier performance and mechanical integrity. These materials not only suppress the heat and flame propagation but also minimize smoke generation, enhance char formation, and sometimes impart additional features like corrosion resistance or conductivity.

In a study conducted by Murtaza et al., an epoxy-based nanocomposite was developed that incorporated a novel hybrid nanofiller made from a 8-hydroxyquinoline (8-HQ)-intercalated CaAl-layered double hydroxide (CaAl-8HQ-LDH) anchored onto reduced graphene oxide (rGO). This multifunctional filler demonstrated an excellent compatibility with the epoxy matrix, ensuring uniform dispersion and strong interfacial adhesion. The resulting coating achieved a UL-94 V-0 flame retardancy rating and exhibited long-term durability in saline conditions, due to the synergistic effect of rGO’s physical barrier function and 8-HQ’s corrosion-inhibiting capabilities. These findings emphasize the dual benefits of fire protection and an anticorrosive performance through nanostructure engineering [105]. The study by Panda et al. presents a high-performance FR and an electromagnetic interference (EMI) shielding system based on carbon nanomaterials. The coating formulation incorporates carbon nanodots, CNTs, and graphene nanosheets within a sodium metasilicate–gypsum matrix. When applied to wooden substrates, the carbon composite coatings exhibit an exceptional flame resistance, effectively minimizing flame propagation and charring during fire exposure at 1050 °C. A thermogravimetric analysis confirms its high thermal stability, with substantial char residue retained at 800 °C, indicating strong thermal insulation. In addition to fire protection, the coating demonstrates significant electromagnetic shielding capabilities, reducing electric and magnetic fields by approximately 50% and 44%, respectively. This EMI performance is attributed to the synergistic effect of carbon nanomaterials, which enhance the conductivity, dielectric loss, and electromagnetic wave absorption. Overall, the study highlights the potential of carbon-based nanocomposites as multifunctional coatings for advanced construction applications, combining a superior flame retardancy with efficient EMI shielding [106]. Moreover, the study has been extended by Panda et al., where a novel FR and EMI shielding coating system was developed using a hybrid nanocomposite approach. The system consists of two-dimensional (2D) hexagonal boron nitride (h-BN) sheets and zero-dimensional (0D) ZnO nanoparticles, integrated into a sodium metasilicate and gypsum matrix. When applied to wooden and wallpaper substrates, these nanocomposite coatings exhibited enhanced flame retardancy, evidenced by the reduced flame spread and carbonization at high temperatures (1050 °C), and excellent thermal stability, with 83.5% of the char residue retained at 800 °C. The coatings also demonstrated effective EMI shielding, particularly at an h-BN:ZnO ratio of 3:1, due to improved resistance and dielectric loss characteristics. Overall, the architectural synergy between h-BN and ZnO contributes significantly to thermal insulation and electromagnetic absorption, making this nanocomposite system highly promising for contemporary building applications [107].

**Figure 13:**
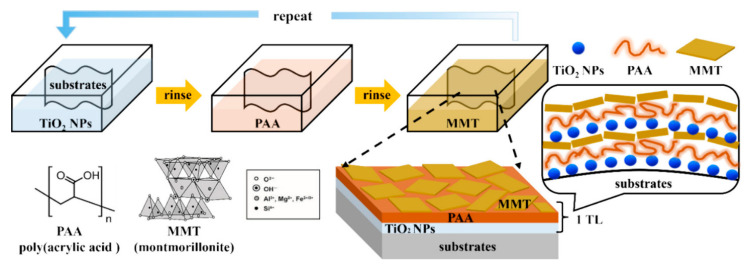
A schematic representation of the LbL assembly, consisting of TiO_2_ nanoparticles, PAA, and MMT, along with chemical structures used [108]. Copyright 2024 American Chemical Society.

In another recent study, Lee et al. synthesized a trilayer (TL) nanocomposite-based fire-resistant coating comprising TiO_2_, PAA, and MMT, which was fabricated via an LbL self-assembly process (Figure 13). The interposition of PAA between the inorganic TiO_2_ and MMT layers provided excellent interfacial compatibility through its carboxylic acid groups, facilitating strong hydrogen bonding and electrostatic interactions. This architecture yielded a highly uniform, thick, and well-integrated nano brick wall structure. The coatings exhibited exceptional thermal insulation and fire retardancy by inhibiting heat flow and promoting dense char formation. The system proved to be highly effective as a protective coating for flammable substrates, offering an eco-friendly, halogen-free alternative for advanced fire safety applications [108]. While nanocomposite-based FRs demonstrate an excellent performance in thermal stability, char retention, and multifunctionality, several trade-offs must be critically considered for real-world applications. The cost remains a major barrier, particularly with carbon nanomaterials such as CNTs, GO, and rGO, which involve complex synthesis processes and high raw material expenses. In terms of scalability, the uniform dispersion of nano-additives at a large scale remains challenging due to agglomeration tendencies and high surface energy, often requiring additional surface treatments or compatibilizers that can complicate manufacturing. Furthermore, the environmental and health impacts of nanomaterials, including concerns related to bioaccumulation, inhalation risks during handling, and long-term degradation, are still under investigation, requiring more extensive regulatory evaluation. Thus, although these materials offer an excellent multifunctional performance, their widespread industrial adoption depends on overcoming cost, processability, and sustainability challenges [109,110].

## 4. Bio-Based and Eco-Friendly Flame Retardants

Bio-based FRs offer a sustainable alternative to traditional halogenated systems by employing renewable raw materials, omitting toxic additives, and adhering to the principles of green chemistry. These systems frequently exhibit excellent flame retardancy while providing enhanced biodegradability, a lower toxicity, and a diminished environmental impact. Various natural compounds, such as TA, PA, lignin, and bio-derived polyphenols, are incorporated into coating matrices for a range of substrates like textiles, wood, and polymers.

Kulkarni et al. developed a green fire-resistant coating for nylon–cotton fabric through a two-step surface functionalization using TA and PA. TA formed hydrogen bonds with nylon, while PA covalently bonded to cotton, creating a synergistic FR effect without requiring synthetic intermediates. The coating adhered to green chemistry guidelines by employing biodegradable resources, omitting halogenated components, and using water-based processing. Although effective in minimizing the heat release and smoke during combustion, its diminished durability after washing and sensitivity to water hardness remain limitations [111].

**Figure 14:**
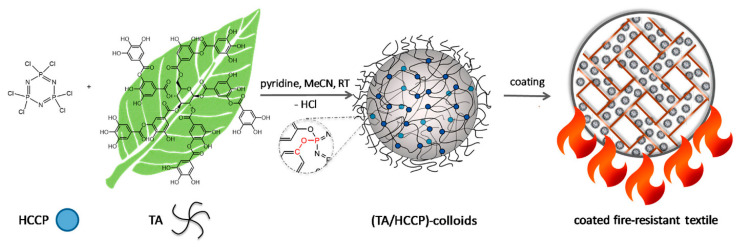
Synthesis route to bio-based (TA/HCCP) colloidal particles [113]. Copyright 2020 American Chemical Society.

Kim et al. synthesized a fire-resistant epoxy resin using TA as a multifunctional biobased hardener, directly reacting with DGEBA. The system achieved flame retardancy through radical quenching and char formation, without the use of halogenated additives. Ethanol served as a low-toxicity solvent, reinforcing the eco-friendly approach. The resulting resin exhibited a higher LOI and lower THR due to its thermally stable carbonaceous structure, highlighting the environmental and functional advantages of TA-based systems [112]. Deniz et al. synthesized a partially bio-based FR by combining hexachlorocyclophosphazene (HCCP) with TA through precipitation polycondensation (Figure 14). The resulting TA/HCCP colloids displayed an excellent thermal stability, strong char-forming ability, and good adhesion to cotton fibers. The coatings achieved LOI values of up to 35 and passed vertical flammability tests. The process employed an aqueous, solvent-free synthesis at room temperature and featured a chlorine content reduction to just 0.08 wt%, underscoring its green chemistry credentials [113].

**Figure 15:**
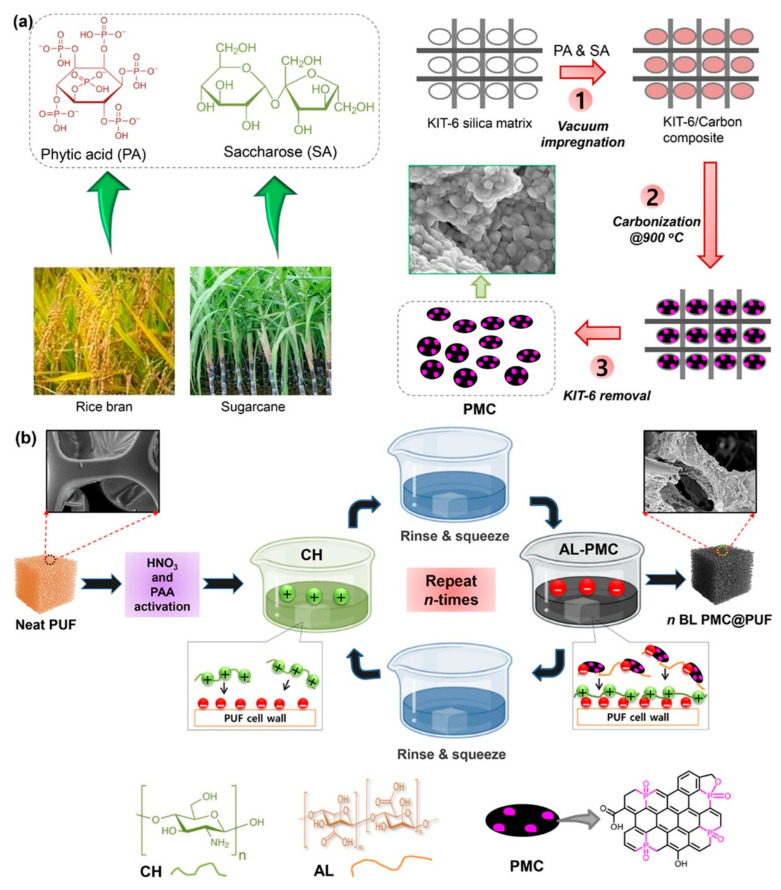
(**a**) The synthesis procedure for PMC and (**b**) the schematic of the LbL assembly approach [114]. Copyright 2022 American Chemical Society.

Weldemhret et al. created a phosphorus-doped mesoporous carbon (PMC) coating from saccharose and PA, using KIT-6 SiO_2_ as a templating agent (Figure 15). This bio-derived, halogen-free material was integrated onto PU foam through an LbL technique that employed alginate and CS as dispersants. With just three bilayers, the coating diminished the PHRR by 56% and reduced carbon monoxide (CO) and carbon dioxide (CO_2_) emissions by 35%. It also demonstrated durability under mechanical stress and adhered to green chemistry principles by utilizing a low energy input, biodegradability, and biomass-sourced feedstocks [114].

**Figure 16:**
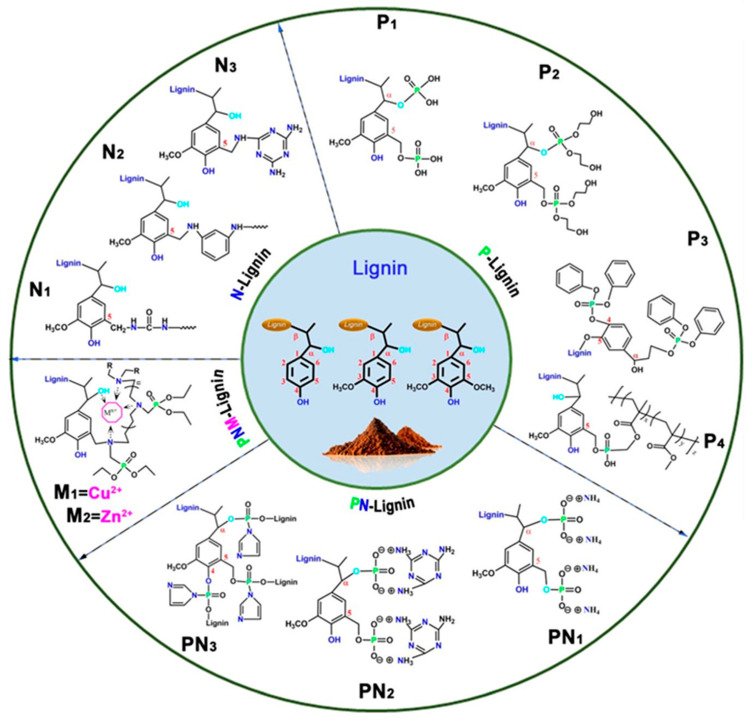
Lignin-derived modified FRs [116]. Copyright 2020 Royal Society of Chemistry.

Zhao et al. designed a bio-based hydrogel fire-resistant coating using polyvinyl alcohol (PVA) and PA, which was applied to wood via a freeze–thaw process in water. The halogen-free, solvent-free formulation relied on hydrogen bonding and char formation to provide fire resistance. The coating increased the time to ignition from 63 s to 130 s and reduced the THR by over 50%. It exhibited strong thermal insulation and a long service life, offering a safe and environmentally sustainable solution for wood protection [115]. Figure 16 represents some lignin-derived FRs modified by phosphorus and nitrogen. Table 2 presents a list of bio-based FRs.

## 5. Functional and Smart Flame-Retardant Coatings

### 5.1. Self-Healing Flame-Retardant Coatings

**Figure 17:**
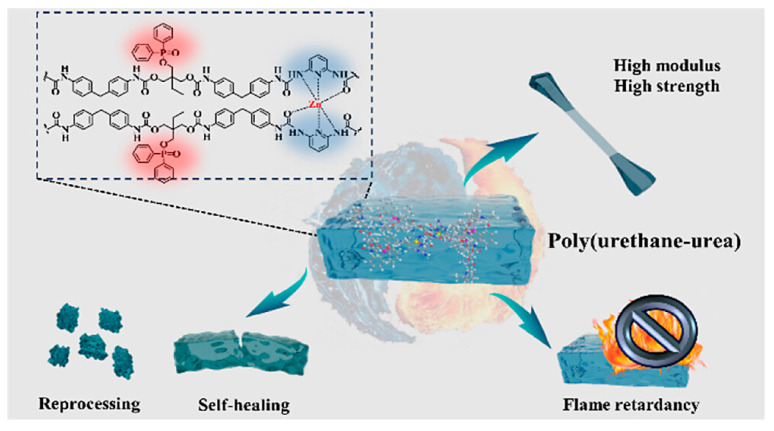
Self-healing poly(urethane–urea) elastomer with good flame retardancy [135]. Copyright 2024 American Chemical Society.

There are two types of self-healing polymer materials based on their mechanisms, namely extrinsic and intrinsic [133]. Extrinsic self-healing polymers require external agents, typically stored in tiny containers like microcapsules within the material. When a crack forms, the healing liquid is released, initiating the repair process. On the other hand, intrinsic self-healing polymers can heal themselves through their own chemical bonds, without any external agent; their structure can be activated by temperature changes, UV light, moisture, or air to start the self-healing process [134]. Luo et al. developed a special poly(urethane–urea) elastomer named PNPU-2%Zn that cleverly combines both self-healing and FR capabilities. Its self-healing occurs through reversible metal–ligand bonds formed between zinc ions (Zn^2+^) and 2,6-diaminopyridine (DAP). These bonds can break and reform when gently heated (around 80 °C), allowing the material to heal itself with an approximately 98% efficiency and no need for high-pressure or complex conditions. To enhance the material’s fire resistance, the researchers incorporated a phosphinate-based chain extender, 2,2-Bis(hydroxymethyl)butyl Diphenylphosphinate. This aids in two ways: in the gas phase, it releases PO• radicals that trap flame-spreading radicals, while in the solid phase, it contributes to the formation of a stable char layer with the assistance of Zn^2+^, preventing the material from burning. Moreover, the nitrogen in DAP produces NH_3_ gas, which does not burn and further aids in stopping flames. Thanks to these combined effects, the material achieves an LOI of 26.5%, passes the UL-94 V-2 flame test, and reduces the heat release by 10%. With excellent strength (20.9 MPa), self-healing properties, and fire resistance, PNPU-2%Zn shows great potential for coatings in flexible and wearable electronics. Figure 17 illustrates the design evolution of self-healing fire-resistant PUs through supramolecular and coordination crosslinks. The incorporation of phosphorus-containing FRs and metal–ligand interactions enhances the flame resistance, mechanical strength, and reprocessability [135].

Meng et al. used a polyelectrolyte complex consisting of PEI and APP combined with GO to create a self-healing fire-resistant coating. They then employed a straightforward dip-nip method to apply the coating to a flexible PU foam. The coating also demonstrated remarkable self-healing behavior: after being damaged, it was able to fully recover within 3 days in air at 50% humidity. As the coating remained soft at room temperature (T_g_ = 16.5 °C), this healing was facilitated by hydrogen bonding and the flexibility of the polymer chains. Additionally, the foam’s mechanical strength increased significantly, showing an excellent performance even after 200 compression cycles and a 175% increase in compressive strength. All things considered, this environmentally friendly coating provides PU foams used in practical applications with durability and fire safety [136]. Sun et al. synthesized a self-healing waterborne plywood coating resistant to fire. Using the semi-interpenetrating network process, they produced the coating by mixing PVA, APP, sodium silicate, and SFS. APP served as the acid and gas source for the flame retardancy, while PVA functioned as the self-healing agent and binder. A denser inorganic silicate network, formed with the assistance of sodium silicate and SFS, enhanced the thermal stability. The PVA/SSA3 formula coating exhibited an excellent fire performance; the fire resistance time improved by 85%, increasing from 114 s (for pure plywood) to 200 s. The char residue at 600 °C also rose to 45.1%, compared to 0% for plain PVA. This robust char layer, formed by APP and the silicate network, helped block heat and oxygen. The coating also demonstrated an excellent self-healing ability when scratched, with marks completely vanishing after a few minutes in water vapor and 30 min at room temperature. This is made possible by PVA chain hydrogen bonding and the water softening effect, which allows polymer chains to move and re-bond. This cost-effective and environmentally friendly coating procedure provides a practical solution for wood protection against both fire and surface damage [137].

### 5.2. Coatings with Dual Functions

Coatings that are resistant to both fire and bacteria are particularly useful in settings where safety and cleanliness are crucial, such as public spaces or hospitals. In addition to being highly effective, these coatings are also safer for both people and the environment because they utilize natural ingredients like CS and ammonium phytate (AP). An environmentally friendly coating made from CS and AP was recently developed by Li et al. to impart both antimicrobial and FR properties to viscose fabric. Using an LbL deposition method, a protective intumescent film was formed on the fabric’s surface. During flame tests, the coated fabric self-extinguished and reached an LOI of 29%, largely due to the CS/AP system forming a char layer that blocked the heat and oxygen transfer. It also released significantly less heat and smoke, with a PHRR of 70 kW/m^2^ and a TSP of just 0.3 m^2^. Besides its ability to withstand fire, the coating demonstrated potent antibacterial properties, reducing S. aureus and E. coli by 99.99%. CS, which attaches to the negatively charged bacterial cell and surfaces and disrupts their membranes, is responsible for this strong antibacterial action. All things considered, this straightforward and environmentally friendly coating has numerous advantages and is a wise option for safer, cleaner textiles [138]. Flame resistance, UV protection, and water repellency are highly sought-after properties for technical textiles used in outdoor gear, industrial safety, and aerospace. Polyimide (PI)-coated fabrics have demonstrated exceptional multifunctionality in meeting these demands. In the study by Hicyilmaz et al., polyamic acid was synthesized and applied to woven cotton and polyester fabrics using a straightforward padding technique, followed by low-temperature iridization to produce PI coatings. In flame tests based on the modified UL-94 standard, PI-coated samples formed a stable ~12 cm char layer that protected the fabric structure and prevented ignition or dripping, whereas uncoated fabrics burned completely and even ignited adjacent materials. Notably, PI-coated polyester did not melt or ignite the cotton indicator, demonstrating outstanding flame resistance. This performance was attributed to the formation of an insulating char barrier by the PI layer, which effectively blocked the heat and oxygen transfer during combustion. Beyond fire resistance, PI coatings provided a robust UV-A protection, reducing the light transmittance from approximately 29% to just 2% at 400 nm. The hydrophobicity also improved significantly, with contact angles reaching 111.43° for cotton and 113.40° for polyester, making the fabrics strongly water-repellent. Young’s modulus of the PI-coated polyester increased fourfold, from 98.85 MPa to 396.49 MPa, indicating an enhanced stiffness and mechanical strength. Overall, this scalable and efficient coating method converted low-cost textiles into high-performance materials suitable for use in demanding, multi-hazard environments [139]. Table 3 shows some examples of FRs used in smart coatings.

## 6. Processing and Application Techniques

### 6.1. Layer-by-Layer Assembly

**Figure 18:**
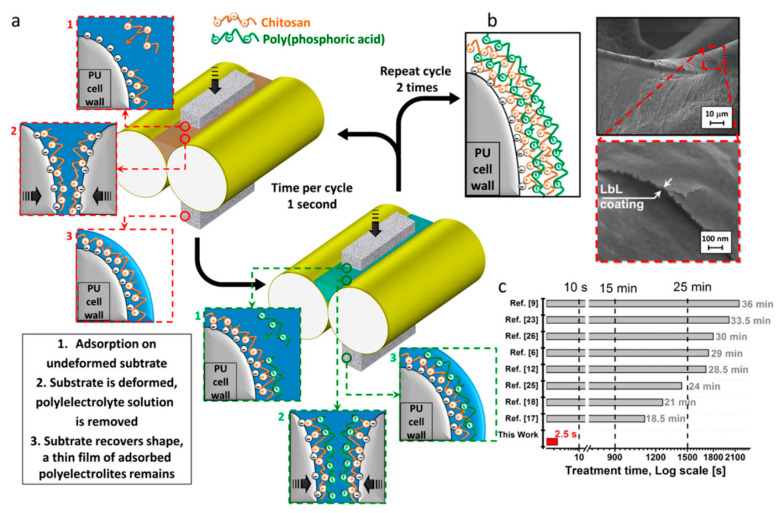
The fast LbL deposition on flexible PU foams: (**a**) the schematization of the process and phenomena occurring during the LbL assembly on flexible PU foam, (**b**) the schematization of 2 bilayers’ dried coating and field emission SEM pictures of 2 bilayer-treated foam, (**c**) a comparison between overall treatment times reported in the scientific literature for the LbL method on PU foams [162]. Copyright 2016 American Chemical Society.

The LbL assembly is a simple, yet powerful technique used to build thin, multilayered films by alternately depositing positively and negatively charged materials, such as polymers, nanoparticles, or biomolecules, onto a surface. The process mainly relies on electrostatic interactions, but other forces like hydrogen bonding or even covalent bonding can also be involved in the materials use [10,161]. One study performed by Carosio et al. developed an ultra-fast LbL technique to apply fire-resistant coatings onto flexible PU foams in just 2.5 s, which is significantly faster than traditional LbL methods. In their process, CS and poly (phosphoric acid) (PPA) are applied alternately using a padder system. The foam is dipped into each solution for only 0.5 s and then immediately compressed between rollers to remove the excess liquid. This quick squeezing step replaces the need for rinses or drying, making the coating process much more efficient. Despite the rapid processing, the method ensures consistent coverage throughout the foam’s complex porous structure. With just two bilayers, the coated foam achieved a 33% reduction in the PHRR, showing an excellent FR performance using a minimal amount of material. By overcoming the usual challenges of long processing times and water retention, this approach provides a practical and scalable solution for improving fire safety in flexible and porous materials. This coating process is displayed in Figure 18 [162].

Yan et al. developed a simple and effective fire-resistant coating for wood using an LbL process. In this method, the wood is repeatedly dipped into two water-based solutions, one containing CS and GO and the other containing APP. These oppositely charged materials bond LbL, creating a protective coating on the wood surface. After applying 15 bilayers, the wood shows a significant improvement in fire resistance. The LOI, which measures the minimum oxygen concentration required to sustain combustion, increased from 22% for untreated wood to 42% for coated wood. Since normal air only contains about 21% oxygen, the coated wood became significantly harder to ignite. In flame tests, the treated wood nearly self-extinguished after 60 s. Cone calorimeter tests indicated a reduction in the PHRR from 207.2 to 162.9 kW/m^2^, and the THR from 62.4 to 34.3 MJ/m^2^. This strong FR effect arises from the combined actions of APP (which releases acid and gas to promote char formation), CS (which strengthens the char), and GO (which serves as a heat and oxygen barrier). Even after 24 h in hot water, acid, or acetone, the coating remained durable and effective, demonstrating its capability in real-world conditions [163].

### 6.2. Sol–Gel Coating Technique

The sol–gel process is a versatile technique employed to create thin coatings with unique surface properties by forming an organic–inorganic network. It commences with liquid precursors, typically metal alkoxides, which undergo hydrolysis and condensation reactions, gradually transforming into a solid gel-like structure [164]. Jiang et al. discovered an ingenious method to utilize a unique coating produced via the sol–gel approach to enhance the fire resistance of polyester fabric significantly. They initiated the process by combining specific chemicals to form a soft silicone gel, which they subsequently applied to the fabric in three layers: a layer of silicone (also known as polysiloxane), followed by a layer of PA, and finally another layer of silicone. The silicone layer acted as a barrier that excluded heat and air, while PA aided in extinguishing the fire by trapping the burning particles. The results were remarkable. The coated fabric ceased burning autonomously and did not drip during fire tests, whereas the untreated fabric ignited rapidly and melted. The fabric also became more resistant to ignition: the LOI value increased from 21.6% to 31.4%, and the burning rate decreased from 1.19 mm/s to 0.32 mm/s. In another test, the heat released during combustion reduced by 65% (from 236.6 to 82.1 kW/m^2^), and smoke was diminished by 72%. Even after washing the fabric 45 times, the coating retained its effectiveness. This demonstrates that employing the sol–gel process alongside PA can render fabrics significantly more fire-resistant, durable, and suitable for practical applications [165].

**Figure 19:**
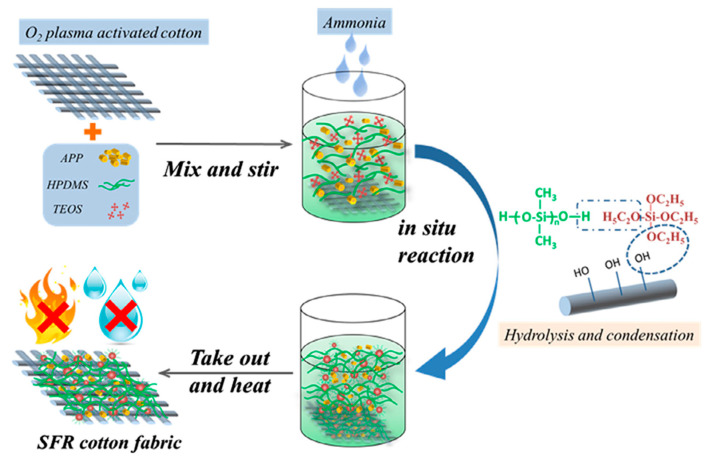
The one-pot fabrication process of the superhydrophobic and fire-resistant (SFR) coating on the cotton fabric [166]. Copyright 2018 Elsevier.

Lin et al. employed a one-step sol–gel method to render cotton fabric both waterproof and fire-resistant (Figure 19). They initially treated the fabric with oxygen plasma and subsequently soaked it in a solution containing tetraethoxysilane, hydroxyl-terminated PDMS, and APP. APP adhered to the cotton fibers through hydrogen bonding, and a sol–gel reaction formed a distinctive PDMS–silica coating on the surface. This produced a rough, micro-, and nano-sized layer that endowed the fabric with excellent water repellency, achieving a water contact angle of 162°, signifying that water rolled off effortlessly. In fire tests, the coated fabric demonstrated remarkable flame resistance, forming a thick protective char layer that inhibited heat and oxygen penetration. When compared to untreated cotton, the coated fabric left significantly more solid residue after combustion, increasing from just 1.4% to 24%, thereby illustrating the efficacy of the coating in protecting the fabric. The peak heat released during combustion decreased by 71%, from 278.8 W/g to 80.6 W/g, while the THR also diminished by 67%, from 9.5 to 3.1 kJ/g. This indicates that the fabric burned less intensely and for a shorter duration. Even following the fire exposure, the coated fabric remained largely intact. Thanks to the synergistic action of the APP (which releases flame-suppressing gases) and PDMS–silica (which forms a heat shield), this coating provides a robust, long-lasting solution for enhancing the safety and durability of cotton fabric in practical applications [166].

### 6.3. Spray, Dip, and Spin Coating Methods

**Spray coating methods**

**Figure 20:**
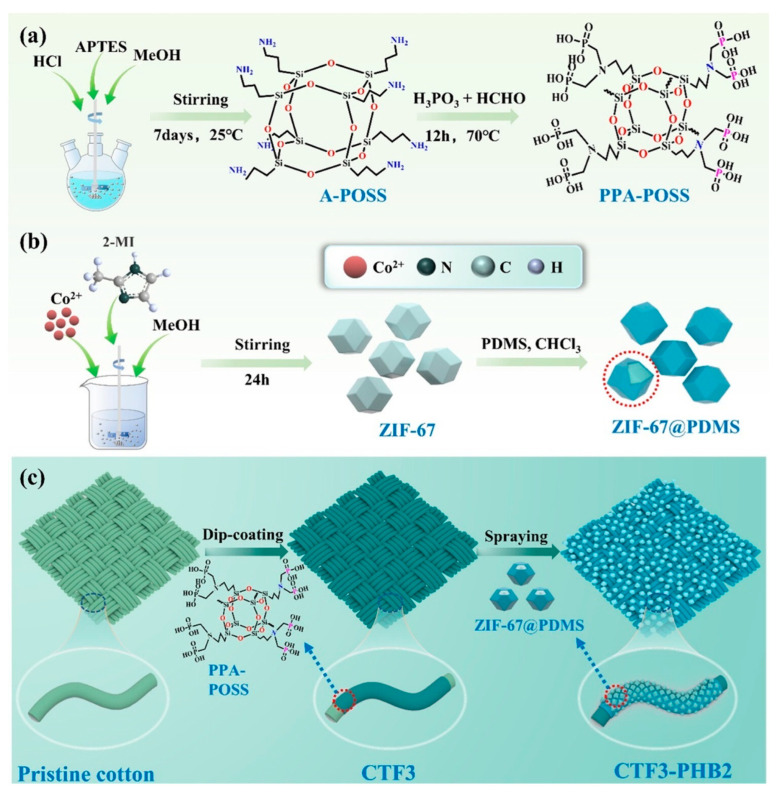
Synthetic routes of the (**a**) PPA–polyhedral oligomeric silsesquioxane (POSS) and (**b**) ZIF-67@PDMS prepolymer; (**c**) a schematic illustration of fabricating the FR and hydrophobic coatings on a cotton fabric [167]. Copyright 2022 American Chemical Society.

Spray coating is an easy and practical method for adding protective layers to fabrics. Liang and colleagues employed this technique to apply a special water-repellent layer made of ZIF-67 nanoparticles mixed with PDMS onto cotton that had been treated to resist fire (Figure 20). They sprayed this mixture onto both sides of the fabric from approximately 15–25 cm and subsequently heated it at 80 °C for 2 h to ensure a strong adhesion. This process resulted in a bumpy micro–nano surface, resembling the texture of a lotus leaf, which helped prevent water from soaking in. Consequently, the coated fabric (CTF3-PHB2) exhibited excellent water repellency, with a water contact angle of 159.3°, and a strong flame resistance, with an LOI of 32%. Even after subjecting the surface to 30 rubs in abrasion tests, the fabric maintained a contact angle above 150°, demonstrating the durability of the coating. This method proved effective without compromising the fabric’s softness or flexibility, making it a smart choice for producing fire-safe, waterproof, and self-cleaning textiles [167].

**Dip-coating method**

**Figure 21:**
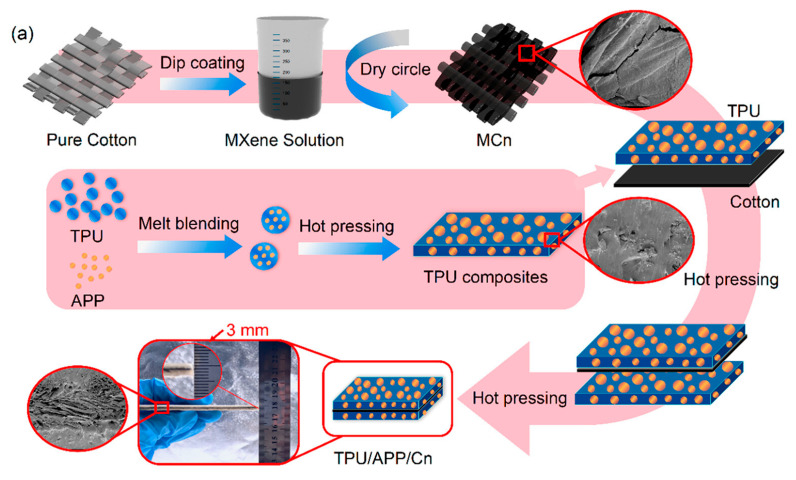
A schematic of the fabrication of the cotton/thermoplastic PU hierarchical composites [168]. Copyright 2023 American Chemical Society.

Dip-coating is a straightforward and effective technique for applying FR materials to fabrics. In the study by Nie et al., the cotton fabric (10 × 10 cm, ~1.80 g) was first cleaned ultrasonically with ethanol and deionized water, then dried in a vacuum oven at 70 °C for 24 h. After that, the cotton was immersed in a Ti_3_C_2_T_x_ dispersion (2 mg/mL) with ultrasonic stirring for 1 h, followed by drying at 60 °C. This dipping process was repeated up to three times, and the samples were designated as MC1, MC2, and MC3 according to the number of coating cycles. With each additional coating, the MXene layer on the cotton surface became thicker and more uniform. SEM images clearly demonstrated that the gaps between cotton fibers were progressively filled with each dip, and after the third coating, the MXene coverage was nearly complete (Figure 21). The TGA in Argon revealed that the char residue increased from 10.36 wt% in pure cotton to 15.62 wt.% in MC3, confirming that the MXene coating enhanced the thermal stability. This dip-coating process enabled easy control over the thickness of the FR layer while maintaining the flexibility of the cotton [168].

Liu et al. developed a fire-resistant coating for PU foam using a straightforward dip-nip process, where the foam was immersed and squeezed in a water-based mixture of APP, MMT, and APTES. After drying, this treatment formed a uniform, compact coating on the foam’s surface. The results were impressive: the LOI increased by 50.8%, rising from 18.2% to 27.3%; the PHRR decreased by 80.28%, from 374.56 to 73.86 kW/m^2^; and the TSP was reduced by 66.7%, from 0.66 to 0.22 m^2^. These significant improvements arose from the combined functions of the three components: APP released acid and non-combustible gases, MMT reinforced the coating structure, and APTES contributed to the formation of a robust Si–O–Si network, all working together to create a thick, stable silicon–phosphorus–carbon char layer during combustion. This protective layer impeded heat, reduced oxygen access, and slowed the release of flammable gases, resulting in a foam that is significantly more resistant to fire and smoke [169].

**Spin coating method**

**Figure 22:**
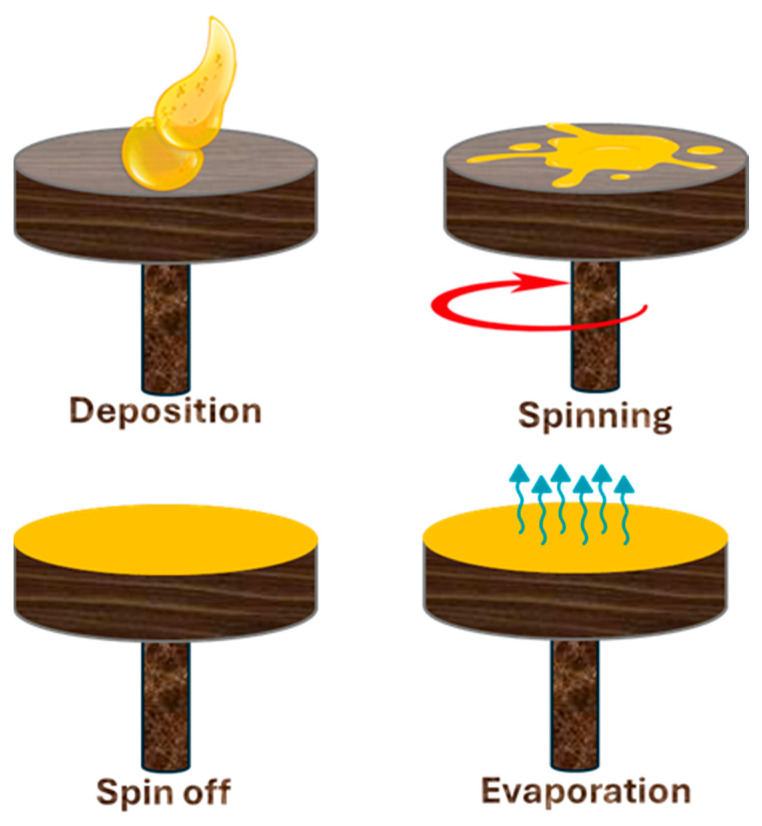
Spin coating process.

Spin coating is a straightforward, yet effective technique widely employed to deposit thin films onto planar substrates. In this process, a small volume of coating liquid is placed at the center of the substrate, which is then spun rapidly, distributing the liquid evenly due to centrifugal force. The spin rate and duration must be meticulously controlled, as even minor variations (±50 RPM) can significantly affect the final film thickness. During spinning, solvent evaporation occurs continuously, leading to the solidification of the coating. The thickness and uniformity of the resulting film are heavily influenced by the solution viscosity, spin speed, and drying conditions, rendering spin coating highly versatile for precise thin film applications [170]. Figure 22 displays the process of spin coating.

Spin coating is a straightforward and efficient method used to create thin and uniform polymer films on surfaces. In this research, the authors employed spin coating to prepare FR nanocomposite films made from PDMS, oxidized multi-walled carbon nanotubes (MWCNT–COOH), and a surfactant. The mixture was spin-coated at 640 rpm and subsequently heat-treated at 120 °C to form solid films. This process facilitated the uniform dispersion of the CNTs throughout the PDMS matrix, resulting in a robust and stable network. The final film, named PDMS/surfactant/MWCNT–COOH (PSM), demonstrated an excellent FR performance. In cone calorimeter tests, the PHRR was reduced by 42%, the SPR decreased by 47%, the TSR was lowered by 18%, CO production fell by 28%, and CO_2_ emissions diminished by 47% compared to the control PDMS. Furthermore, the LOI of PSM was 31.5%, which is significantly higher than the 25.3% of the plain PDMS, indicating that it is more resistant to ignition. These enhancements are attributable to the dense char layer and physical barrier formed by the well-dispersed CNTs, which slow combustion and decrease the smoke and toxic gas release. Spin coating, therefore, proved to be an effective technique for producing high-performance FRs [49].

## 7. Industrial and Real-World Applications

FRs play a crucial role in enhancing fire safety across a wide range of industries. By slowing or preventing the spread of fire, these materials contribute significantly to reducing property damage and saving lives. Their applications span diverse sectors, reflecting their importance in modern safety designs.

### 7.1. Flame-Retardant Coatings in Construction Materials

The construction industry is a pillar of modern society, and the fire resistance of building materials is critical for safeguarding lives and property. Developing polymer products with excellent FR properties for use in structural and interior applications is essential. Commonly used polymers include polyethylene, polypropylene, polystyrene, poly vinyl chloride, phenolic resin, urea–formaldehyde, PU, and epoxy, which are widely applied in walls, ceilings, floors, and cables. However, many of these materials are flammable and fail to meet standard fire safety requirements. To address this, flame retardancy must be improved without compromising the mechanical performance. Ideal materials should offer a strong fire resistance, good thermal insulation, and long-term durability, along with features such as low thermal conductivity and high adhesion. Additionally, an easy application, water resistance, and a faster installation can reduce construction costs while ensuring compliance with national quality standards [171]. Vakhitova et al. developed a high-performance intumescent fire-resistant coating to protect steel structural elements in buildings. The coating operates on an intumescent mechanism, where an exposure to fire causes it to expand and form a dense char layer that insulates the surface, slows heat transfer, and preserves the mechanical integrity of the steel. It was formulated using a reactive system consisting of APP, MA, and PER, combined with carefully selected polymer binders. Among these, vinyl acetate–ethylene–vinyl versatile copolymers (e.g., EZ 3112) demonstrated the highest efficiency, producing a char with an expansion volume of 45 cm^3^/g and maintaining structural protection for up to 62 min at a dry film thickness of 1. 1.5 mm. In comparison, solvent-borne styrene acrylate systems (e.g., AC 80) showed a lower FR performance, with their fire resistance reduced to 32 min under the same conditions. Optimizing the APP:MA:PE ratio to approximately 3.5:1:1.5 resulted in a more compact char structure and enhanced protection. To further improve the flame resistance and mechanical strength, nano-clay additives such as Garamite 7305 were incorporated at 0. 0.3–0.6%, increasing the fire rating by 10–12 min and minimizing the degradation after 300 days in humid conditions. Glass and mineral fiber reinforcements were also employed to stabilize thicker coatings, preventing char detachment during extended fire exposure. Through this combined formulation strategy, the final coatings achieved fire protection durations of up to 120 min, demonstrating their effectiveness and scalability for passive FR applications in steel-based construction systems [172].

### 7.2. Automotive and Aerospace Coating Applications

Fire-resistant coatings play a critical role in enhancing fire safety in transportation sectors, such as automotive and aerospace industries, where high-performance polymers are widely used due to their mechanical strength, lightweight nature, and design flexibility. However, many of these materials, such as polyamide 6 (PA6), are inherently flammable and exhibit melt-dripping behavior during combustion, posing serious fire hazards. Developing environmentally friendly, high-efficiency fire-resistant coatings is therefore essential for improving the safety of these materials without compromising their mechanical integrity or adding toxic components.

In this regard, Liu et al. introduced a fully bio-based fire-resistant coating composed of TA and TN, which was applied to PA6 fabric using a scalable and industry-relevant dip-nip technique. PA6, commonly used in automobile interiors, military textiles, and aircraft seating, was rendered significantly safer with this coating. The enhancements in flame retardancy were remarkable: the LOI increased from 19.4% to 26.8%, and the fabric achieved the highest UL-94 V-0 rating, exhibiting no dripping and a reduced burn length of 4.5 cm. An advanced cone calorimeter analysis further confirmed the coating’s efficacy. The PHRR dropped by 20% (from 502 to 402 kW/m^2^), the THR decreased by 31.3%, and the smoke production was reduced by an impressive 66.7%. Mechanistically, the bio-coating provided dual-action protection: TA facilitated the formation of a char layer, serving as a barrier to heat and oxygen, while TN released non-flammable sulfur dioxide gas, effectively suppressing flame propagation and smoke. This innovative and eco-friendly coating system not only complies with green chemistry principles by using renewable, non-toxic, and biodegradable ingredients, but also offers practical fire protection for synthetic fabrics in critical applications. It holds strong potential for integration into next-generation fire safety protocols across the automotive, defense, and aerospace industries [132].

### 7.3. Textile and Paper Coatings

Textile and paper-based materials are inherently flammable due to their high cellulose content, rendering them particularly susceptible to ignition and flame propagation. As these materials are extensively utilized in household, industrial, and commercial settings, developing effective fire-resistant coatings is essential to enhance their fire safety. Applying functional surface coatings provides a direct and practical approach to delaying ignition, suppressing heat release, and reducing the flame spread without significantly altering the material’s inherent mechanical or esthetic properties.

In a recent study, Deniz et al. engineered a bio-based fire-resistant coating using colloidal particles composed of TA, a naturally occurring polyphenol, and HCCP, a phosphorus–nitrogen-rich FR. These TA/HCCP colloids were applied to cotton fabrics using a water-based dispersion method, consistent with green processing principles. The coated fabrics exhibited remarkable fire resistance, with the LOI reaching 35 and the char residue improving to 36% under a nitrogen atmosphere. In vertical flame tests, uncoated cotton ignited rapidly, displaying an after-flame time of 32.3 s and an after-glow time of 57.7 s. In contrast, the coated fabric self-extinguished immediately, with no glowing and a minimal char length of just 2–2.5 cm. This superior performance was attributed to the synergistic phosphorus–nitrogen FR mechanism, where HCCP facilitates phosphorus-driven char formation, and TA contributes intumescent behavior and thermal shielding [113]. In another study, Zope et al. developed a spray-applied fire-resistant coating using para-phenylenediamine and tetrakis(hydroxymethyl)phosphonium chloride. These components swiftly formed a polymeric phosphate–nitrogen-rich protective layer on the fabric surface. The treated cotton exhibited after-flame times reduced to just 2–3 s and the complete elimination of after-glow, in stark contrast to the prolonged burning of untreated cotton. Additionally, the char residue increased from 11.5% to 28.6%, and the THR was reduced by approximately 45%, highlighting the coating’s ability to limit the combustion intensity. The fabric maintained its structural integrity even after the flame exposure, without disintegration or dripping. The success of this system lies in the synergistic interaction of phosphorus and nitrogen, which catalyzes the char formation and creates a stable insulating layer during combustion. These advanced fire-resistant coatings not only offer high fire resistance but also align with environmental safety and practical applicability. They hold immense potential for high-risk textile applications, such as firefighter uniforms, military clothing, laboratory coats, and racing gear, where fire protection is essential without compromising flexibility and wearability [173].

### 7.4. Electronic and Cable Coatings

In modern electronics, fire safety and thermal management are critical concerns due to the increasing integration of compact and high-energy components, such as lithium-ion batteries and flexible circuits. Cables, connectors, and devices must be protected against overheating, short circuits, and potential flame hazards, particularly in confined spaces like consumer electronics, electric vehicles, and data centers. Advanced fire-resistant coatings not only serve as thermal insulators but also suppress combustion, delay heat propagation, and improve the overall device safety without compromising the electrical performance or flexibility.

Yu et al. engineered an innovative foamy aerogel coating composed of a melamine–formaldehyde resin integrated with SiO_2_ aerogel particles, specifically designed for electronic insulation and thermal shielding. The coating demonstrates an ultralow thermal conductivity, as low as 0. 027 W·m^−1^·K^−1^; a high porosity; and an LOI of 33%, significantly surpassing traditional polymer insulators like PU and polystyrene. Its unique hierarchical porous structure prevents resin intrusion while maintaining superior insulation. Critically, in lithium battery needle penetration tests, the coating reduced the surface temperature by over 200 °C, showcasing its ability to suppress thermal runaway events. Additionally, it exhibits strong adhesion and water resistance, and is compatible with air-spray applications, making it scalable for industrial use. This study establishes the aerogel system as a cost-effective and high-performance material for fire-safe and thermally managed electronic applications, particularly in energy storage devices and battery modules [174]. In another development, Zhang et al. designed a biomimetic nanoporous transparent fire-resistant coating using a one-pot sol–gel synthesis approach. The precursor system combined phosphorous acid (H_3_PO_3_), N-[3-(trimethoxysilyl)propyl]ethylenediamine (KH-792), and dimethoxydimethylsilane to yield a P/n/si-based hybrid polysiloxane network. The resulting coating achieved a high optical transparency (>97%), a superior adhesion to various substrates, and an LOI of 33.5%. Notably, the PHRR and THR were reduced by 59.8% and 48.4%, respectively, confirming their excellent flame retardancy. Its flexibility, breathability, and mechanical robustness make it ideal for flexible and wearable electronics, such as smart textiles, medical sensors, and soft robotics. During combustion, the coating forms a dense, graphitized char layer that serves as a thermal barrier, effectively suppressing flammable gas emissions and blocking oxygen diffusion, which collectively prevent fire propagation. Together, these coatings represent a new class of high-efficiency, multifunctional FR materials that combine the thermal insulation, fire suppression, and mechanical resilience essential for next-generation electronic packaging, power storage, and smart wearable systems [175].

## 8. Challenges and Future Perspectives

### 8.1. Durability, Weatherability, and Aging of Fire-Resistant Coatings

One of the most pressing challenges in the practical implementation of fire-resistant coatings is ensuring their long-term durability, particularly under harsh environmental conditions. These coatings must consistently retain their FR efficacy despite their exposure to mechanical stress, humidity, UV radiation, and fluctuating temperatures. While bio-based FR systems offer significant environmental advantages, they often exhibit hydrolytic instability and a reduced performance when subjected to external stressors, such as washing or prolonged weathering. For example, coatings formulated with natural compounds like PA or TA demonstrate a good initial fire resistance but degrade rapidly upon exposures to moisture and laundering cycles, compromising their long-term protective function [111]. This issue is particularly pronounced in textile applications, where repeated washing and mechanical abrasion can deteriorate both the char-forming agents and the adhesion between the coating and substrate. The result is a gradual decline in fire resistance, limiting the effectiveness of the coating over time. Similarly, silicone-based coatings, such as those made from PSZs, exhibit an excellent initial flame retardancy due to the inherent stability of their silicon backbones. However, their long-term weatherability is contingent upon the stability of Si–O and Si–N bonds and their interfacial compatibility with the substrate, which may weaken under environmental stress [51]. To overcome these limitations, advanced hybrid FR systems have been developed that incorporate inorganic components like SiO_2_ or TiO_2_ into organic polymer matrices, including acrylics and PUs. These hybrid structures offer an improved thermal stability and resistance to UV degradation, while also enhancing the adhesion and char integrity. Nevertheless, challenges remain in preserving key performance metrics, such as transparency, mechanical flexibility, and structural cohesion, after prolonged aging or environmental exposure [105]. Addressing these durability concerns is essential for translating lab-scale FR technologies into robust, real-world applications.

### 8.2. Toxicity and Regulatory Issues

The toxicological profile of FRs remains a significant focus of regulatory attention due to their potential adverse effects on human health and the environment. HFRs, particularly PBDEs and TBBPA, have been linked to serious health risks, including endocrine disruption, developmental toxicity, and bioaccumulation. These concerns have led to stringent regulatory actions. For example, the European Union’s Registration, Evaluation, Authorisation and Restriction of Chemicals regulation, along with the Restriction of Hazardous Substances Directive, has imposed significant restrictions or outright bans on the use of these compounds in consumer products [7]. As a result of these regulatory pressures, there has been a strong push within the industry to replace halogenated FRs with safer, halogen-free alternatives. Among the most promising substitutes are phosphorus-based compounds, such as DOPO derivatives, as well as nanomaterials like CNTs and GO. These materials have demonstrated a reduced toxicity in short-term toxicity studies and are regarded as more environmentally benign compared to traditional halogenated systems. However, their long-term environmental behavior and safety profiles remain insufficiently characterized, raising concerns about their potential cumulative impacts [45]. Moreover, even FRs marketed as “green” or sustainable are not without issues. Their degradation products, formed during their service life or combustion, can sometimes be equally toxic or persistent, thereby introducing new regulatory challenges. These concerns highlight the necessity of adopting a more holistic approach to FR development—one that is grounded in the principles of green chemistry and includes comprehensive life cycle analyses and toxicity evaluations. Only through such rigorous assessments can FRs be deemed truly safe and sustainable for widespread commercial use.

### 8.3. Toward Halogen-Free and Sustainable Alternatives

The global shift towards halogen-free and environmentally sustainable FRs has gained significant momentum, driven by the increasing awareness of ecological risks and human health concerns associated with traditional halogenated compounds. In this evolving landscape, phosphorus-, nitrogen-, and silicon-based FRs have emerged as leading alternatives, providing effective fire protection while minimizing the environmental impact. Among these, intumescent systems incorporating APP and MA are particularly noteworthy for their high efficiency in both gas-phase inhibition and condensed-phase char formation, all without the use of halogenated species [11]. Phosphorus-containing monomers, such as DOPO and its derivatives, are increasingly being utilized in UV-curable and reactive polymer matrices. These compounds contribute to multifunctional performances by enhancing flame retardancy while simultaneously reducing smoke production and the release of toxic gases during combustion [37]. In parallel, bio-based materials, like lignin, TA, and PA, are gaining popularity for their inherent low toxicity, renewable origin, and excellent char-forming capabilities, making them attractive candidates for sustainable FR formulations [176]. Despite these advancements, several challenges still hinder the widespread adoption of halogen-free FRs. Scalability and cost remain significant barriers, particularly for bio-based systems, which often require complex processing or chemical modification. Furthermore, performance limitations, especially under high-temperature or long-duration fire exposure, continue to pose a hurdle. To address these issues, researchers are actively exploring hybrid systems that integrate bio-based char formers with inorganic reinforcements, such as SiO_2_ or LDHs. These hybrid structures aim to balance environmental compatibility with a robust thermal stability and fire resistance, thereby bridging the gap between ecological safety and high-performance requirements [20].

### 8.4. Research Gaps and Emerging Technologies

Despite significant advancements in fire-resistant coating technologies, several critical research gaps persist, hindering their widespread and effective application. Although numerous studies demonstrate an enhanced fire resistance through standardized laboratory-scale evaluations, such as the LOI and UL-94 vertical burn tests, far fewer investigations assess performances under real-world conditions. These conditions include mechanical abrasion, prolonged UV exposure, and multi-hazard scenarios that simultaneously involve fire, moisture, and physical stress. Such factors can significantly affect the protective performance of coatings, yet they remain largely unaddressed in the current literature [135]. The integration of intelligent functionalities into fire-resistant coatings, such as self-healing behavior, thermochromic responses, or electrical conductivity for early fire detection, also remains underdeveloped, particularly in commercialized systems. While these features offer potential for multifunctional fire safety solutions, they have not yet achieved widespread adoption, partly due to challenges in material compatibility, long-term stability, and processing complexity [177]. Advanced fabrication techniques, such as LbL assembly and sol–gel processes, have shown promising results in producing transparent and highly effective fire-resistant coatings. These methods enable the precise control over the coating architecture and allow for the incorporation of both organic and inorganic FR agents. However, their industrial scalability is constrained by factors like slow processing times, water retention in the coating layers, and limited compatibility with diverse substrates [10]. Emerging technologies in additive manufacturing and 3D-printable coatings present an exciting frontier for developing structure-responsive FR systems. These approaches facilitate the direct incorporation of fire-resistant features into digital designs, allowing for the spatial control over FR properties and the creation of multifunctional parts in a single fabrication step. Nonetheless, these innovations remain in their infancy and require further research to optimize material formulations, printing parameters, and functional performances [178]. Moreover, there is a growing consensus within the research community regarding the need for standardized evaluation protocols that extend beyond traditional thermal performance metrics. Future assessment frameworks should encompass parameters such as the aging behavior, recyclability, environmental footprint, and end-of-life impacts. Such comprehensive evaluation criteria are essential for validating the long-term sustainability, safety, and functionality of next-generation fire-resistant coatings, thereby ensuring their successful transition from laboratory development to real-world implementation.

## 9. Conclusions

In recent years, fire-resistant polymer research has increasingly focused on developing advanced and sustainable approaches to fire safety. HFRs, once ubiquitous for their effectiveness, are now being replaced by safer alternatives such as phosphorus-, nitrogen-, and mineral-based systems due to heightened environmental and health concerns. Simultaneously, the field has embraced nanocomposites and hybrid formulations that combine multiple FR mechanisms at different scales, yielding synergistic improvements in flame suppression, thermal stability, and even mechanical integrity. This evolution reflects a broad trend toward FR materials that achieve a high performance without sacrificing material properties or sustainability goals. These advancements have significant implications for materials design and sustainability. With more efficient and synergistic FR additives, engineers can design polymer systems that meet stringent fire safety standards without compromising on their weight or mechanical performance. The integration of nanoscale reinforcements (such as layered silicates, graphene, or metal oxides) not only enhances flame retardancy but can also improve a polymer’s strength and thermal stability, reducing the trade-offs typically associated with adding FRs. Equally important, the rise of bio-based FRs which are derived from renewable resources, like plant polyphenols, phosphorus-rich biomolecules, and other biomass derivatives, ensures that fire-safe materials are also environmentally responsible, minimizing toxic smoke and persistent pollutants. This alignment of fire safety with eco-friendly design is increasingly critical as industries seek materials that protect both people and the planet. Looking ahead, fire-resistant polymer research is poised to advance further through innovative nanocomposites, hybrid systems, and bio-based strategies. Future FR designs will likely exploit the complementary strengths of multiple additives, for example, combining char-forming biopolymers with inorganic nanofillers to achieve superior protection with a minimal additive load. There is also a growing push toward imparting additional functionality to FR materials, such as self-healing capabilities, thermochromic (temperature-indicating) responses, or even integrated electrical conductivity for smart fire response systems. Realizing these multifunctional fire-resistant polymers will require interdisciplinary collaboration and a careful attention to remaining challenges like scalability, costs, and long-term durability. Addressing these factors will be crucial to ensure that lab-scale breakthroughs translate into reliable, real-world solutions. Ultimately, the future of fire-resistant polymers lies in creating materials that are not only highly fire-resistant but also sustainable and multifaceted in performance. By harnessing nanoscale innovations, synergistic additive combinations, and renewable feedstocks, the next generation of fire-resistant polymer systems will align with ever-stricter safety regulations and global environmental standards. These advancements promise to enable safer buildings, textiles, electronics, and transportation systems. This broader impact positions fire-resistant polymer technology as a key contributor to public safety and sustainable development in the years to come.

## Figures and Tables

**Table 1:** Various FRs and their properties.

Sr. No	Material	Flame Retardant	Mechanical Property	Thermal Property	Flame Retardancy (LOI %)	PHRR (kW/m^2^)	Char Yield (%)	Ref.
1	Polyether polyols, toluene diisocyanate	2-carboxyethyl(phenyl)phosphinic acid (CEPP) and MA	Tensile—0.11 MPa	TGA—383 °C	25.6	240	4.3	[58]
2	Dimethyl/diphenyl phosphoramidates	Tris-(1-chloro-2-propyl) phosphate	-	TGA—407 °C	23.9	-	-	[59]
3	Epon 828	Poly(acrylic acid), APP, MA	-	TGA—270 °C	-	229	-	[60]
4	Silicon wafers, cationic CH-stabilized mica	CS and poly(acrylic acid) (PAA)	-	TGA—600 °C	-	305	-	[61]
5	Epoxy–polyamide (EP)	APP, sodium polyacrylate	-	-	31.3	-	-	[62]
6	Epoxy resin	P/N/Co-containing microsphere FR (FNP-Co)	-	-	30.2	-	-	[63]
7	Polyester fiber	Polydimethylsiloxane (PDMS)	-	-	22.5	-	-	[64]
8	Vanillin, 4,4′-diaminodiphenyl ether	4,4′-diaminodiphenylmethane (DDM)	-	T_g_—188.9 °C	35.2	-	-	[65]
9	Polyester fabrics	6-((((2-(acryloyloxy) ethoxy) (oxo)(phenyl)phosphonio) oxy) methyl) dibenzo[c,e] [1,2]oxaphosphinine 6-oxide and tri(acryloyloxyethyl) phosphate	-	-	24	-	-	[66]
10	Luteolin-derived epoxy resin,	5, 5′-methylenedifurfurylamine	Tensile—66.3 MPa	T_g_—216 °C	38	-	-	[67]
11	Tris(hydroxymethyl)phosphine oxide (THPO), 3,5-diamino-1,2,4-triazole (guanazole)	PDMS	-	-	32	48.2	13.5	[68]
12	Polyelectrolyte complexes	Phosphorus acid, CS	-	-	64.3	-	-	[69]
13	-	Chitooligosaccharide	-	TGA—372 °C	28	380.35	-	[70]
14	Silk fabric	Tannin, humic acid, fulic acid (FA), and green alum	-	-	28.5	-	-	[71]
15	Polyether polyol (ZS-4110), PDMS	DOPO with fumaropimaric acid-based siloxane	Flexural—1.08 MPa	TGA—332.8 °C	26.1	170	-	[72]
16	Polycarbonate diol, isophorone diisocyanate (IPDI)	4-DOPO-((3-hydroxypropyl) imino) methyl)	Tensile—16.6 MPa	TGA—423 °C	28.6	159	12.6	[73]
17	Polyether polyol, methylene diphenyl diisocyanate	Fluorine- and nitrogen-based synthesized FRs	-	TGA—335 °C	19.7	109.8	-	[74]
18	Polylactic acid (PLA) (3001D)	APP and CS and carboxylated silicone oil (Si-COOH/Si)-based FRs	Tensile—53 MPa	TGA—381 °C	34	387	3.1	[75]
19	PLA (4032D)	APP- and CS-based FRs	Tensile—45.3 MPa	TGA—382 °C	30.5	252	14	[76]
20	Poly(ethylene–vinyl acetate)	Brucite/3-aminopropyltriethoxysilane (APTES)/nickel alginate/APTES (B/A/Nia/A)-based FRs	-	TGA—414 °C	32.3	355.	-	[77]
21	Biphenol A (E-44)	APP- and diethylenetriamine-based FRs	-	TGA—329 °C	30	310	66.5	[78]
22	Graphene oxides	Polymer-functionalized graphene composites	Tensile—40.1 MPa	TGA—600 °C	47.6	-	-	[79]
23	Anionic poly(vinylphosphonic acid)	Cationic CS	-	TGA—476 °C	-	76	1	[80]
24	Cotton fabrics	Deoxyribonucleic acid and CS	-	-	24	57	13	[81]
25	Acrylic resin	Al(OH)_3-_ and Mg(OH)_2_-based FRs	-	TGA—750 °C	34	-	-	[11]
26	Expandable polystyrene	Polysiloxane-based FRs	-	TGA—422 °C	36	138.2	-	[82]
27	Cotton fabrics	PA- and polyethylenimine-based FRs	Tensile—28.94 MPa	TGA—290 °C	37	176	-	[83]
28	Cotton fabrics	Phosphorus monomer	-	TGA—220 °C	27	77.4	-	[84]
29	Polyethylene terephthalate fabrics	GP-108- and PA-based FRs	Tensile—575 MPa	TGA—429 °C	27	139	-	[85]
30	Polyethylene glycol borate	Phosphoric acid, n-butyl alcohol, and PER	-	TGA—613 °C	-	116.8	-	[86]
31	Mg(OH)_2_ and fcyclic phosphate ester	Magnesium phosphate ester	-	TGA—361 °C	-	162	26	[87]
32	Branched polyethyleneimine (BPEI)-kaolinite (kaol)-BPEI-diethylene triamine penta (methylene phosphonic acid))	Expandable polystyrene	-	-	37.7	220.9	32.3	[88]
33	Castor oil- and 3-mercaptopropionic acid-based	[(6-oxido-6H-dibenz [c,e] [1,2] oxaphosphorin-6-yl) methyl] butanedioic acid- and allyl glycidyl ether-based FRs	-	TGA—343 °C	27.52	147	14	[89]
34	Cotton fabrics	PDMS	-	TGA—496 °C	29	81	15	[90]
35	Polyester–cotton fabrics	APP	-	TGA—523 °C	-	128	5.9	[91]
36	Branched polyethylenimine	Phenylboronic acid	-	TGA—298 °C	29.6	129	-	[92]
37	Polyester–cotton blend fabrics	CS/PA	-	TGA—443 °C	29.2	195.88	22.84	[93]

TGA—hermogravimetric analysis, T_g_—glass transition temperature.

**Table 2:** Bio-based FRs.

Sr. No	Flame Retardant	Structure	Flame Retardancy (LOI%)	PHRR (kW/m^2^)	Char Yield (%)	Ref.
1	PA	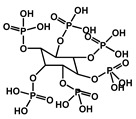	32	-	-	[117]
2	Hexakis (4-aminophenoxy) cyclotriphosphazene-PA with microporous nanosheet	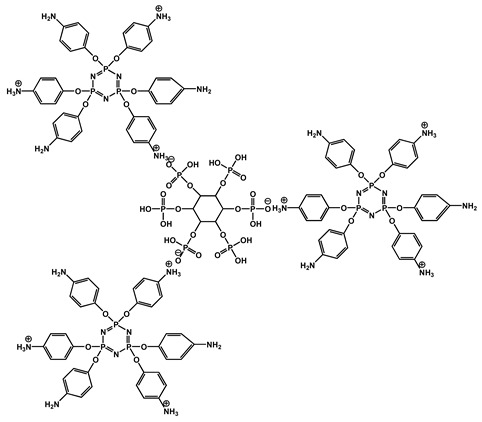	27.3	422.1	12	[118]
3	CS	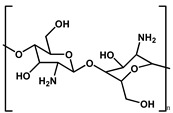	31	66	87	[119]
4	TA	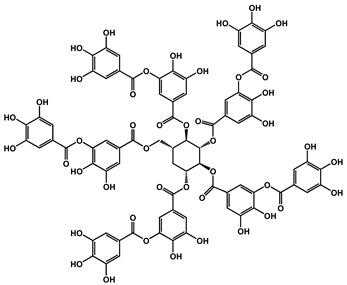	-	730	-	[120]
5	Choline phytate	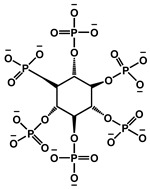	43.7	152.34	-	[121]
6	Imidazole phytate	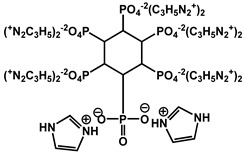	34.7	344	-	[122]
7	Lignin, casein, and PA	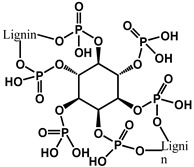	32.5	71.4%	-	[123]
8	PA and furfurylamine	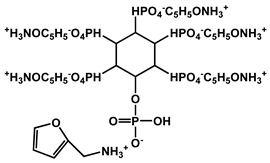	28.5	332.7	-	[124]
9	PA–tyramine salt	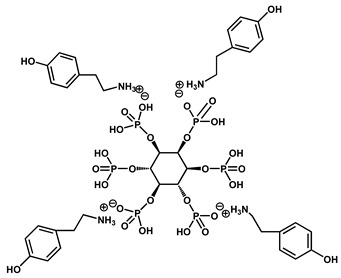	25	310	43.4	[125]
10	PA and Mg(OH)_2_	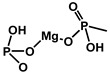	30.8	-	-	[126]
11	PA	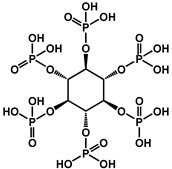	32.2	90.69	-	[127]
12	A novel bio-based FR	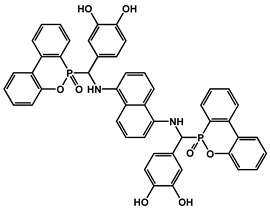	33.6	696.3	17	[128]
13	Phenylphosphorylaminated microcrystal cellulose	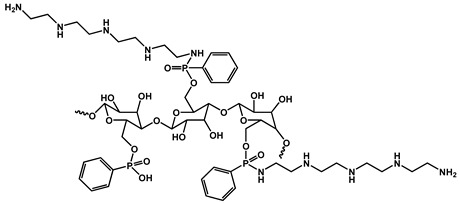	27	563.2	-	[129]
14	CS/melamine formaldehyde-resin-coated APP/MMT	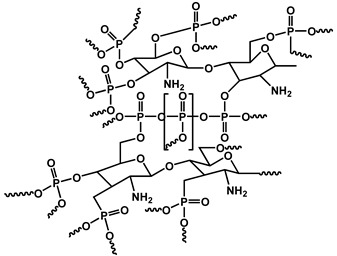	32.0	277.5	35.3	[130]
15	Phosphorus–nitrogen flame retardant	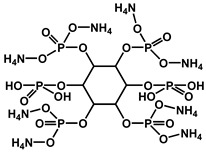	42	-	-	[131]
16	PA	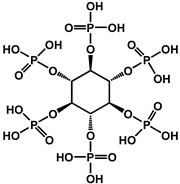	32.3	90.69	-	[127]
17	TA- and PA-based FRs	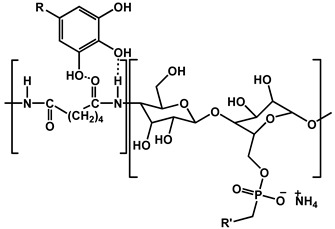	-	167	26.6	[111]
18	PA 6-taurine (TN)/TA	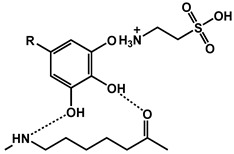	26.8	402	-	[132]

**Table 3:** FRs that are used in smart coatings.

No	Smart Material	FR Name	Mechanical Property	Thermal Property	Flame Retardancy (LOI %)	PHRR (kW/m^2^)	Char Yield (%)	Ref.
1	Polytetra methylene ether glycol, dimethylolpropionic acid (DMPA), IPDI	Di(1-hydroxyethylene) diselenide (DiSe)	Tensile—15.6 MPa elongation 852%	T_max_—365.9 °C T_g_—66.8 °C	28.1	519.22	0	[140]
2	PVA	Sodium fluorosilicate (SFS), APP, sodium silicate	-	T_max_—362 °C	-	-	-	[137]
3	Epoxidized soybean oil, IPDI, DMPA, dimethylglyoxime	DOPO	Tensile—1082 N, elongation—24.1%	T_max_—443.7 °C	22.5	-	-	[141]
4	Gelatin, CS, glycerol	Bio-gel itself acts as an FR	Tensile—1.3 MPa	TGA—glycerol and CS degraded between 170 and 300 °C and gelatin degraded between 250 and 330 °C	-	252.2	-	[142]
5	dPTD (includes (DiSe) and dPTB (1,4-butanediol)	DOPO, isocyanatopropyltriethoxysilane, polyethylenimine	(dPTB-mfGO2) tensile—17.8 MPa elongation—1249.3%, (dPTD-mfGO2) tensile—15.6 MPa elongation—1128%	T_g_ (dPTB-mfGO2)—34.5 °C T_g_ (dPTD-mfGO2)—32.1 °C	24.9	-	-	[143]
6	CS citric acid	Vanillin-based phosphorus-containing epoxy	Tensile—41.2 ± 1.1 MPa elongation—78.8%	T_g_—111 °C	41.2	16	-	[144]
7	Polypropylene glycol, IPDI	Tri(2-furyl) phosphoramide	Tensile—27.89 MPa elongation—827%	T_max_—383.5 °C	28.5	572.5	-	[145]
8	4,4-dithiodianiline, p-hydroxybenzaldehyde benzyl mercaptan, 3-chloroperoxybenzoic acid	HCCP	Impact—18.2 MPa tensile-28.70 MPa	T_g_—129 °C Tmax—335 °C	30.5	-	-	[146]
9	Polyethylene glycol, triethanolamine, IPDI	TBBPA	Tensile—6.37 MPa, elongation—21%	T_max_—320–350 °C	23.6	937.94	7.96	[147]
10	Pentaerythritol tetra(3-mercaptopropionate	4-aminophenyl disulfide	Tensile—1.97 MPa, elongation—85.66%	T_g_—22.62 °C T_max_—296.8 °C	28.36	569.25	0.12	[148]
11	Tris [2-(acryloyloxy)ethyl] isocyanurate	K20 HGM (hollow glass microspheres)	Compressive strength—81.8 ± 7.5 MPa	T_g_—250 °C	-	-	-	[149]
12	Vanillin 1,3-Bis(3-aminopropyl) tetramethyldisiloxane, DDM	-	Tensile— 54.5 ± 1.1 MPa, elongation—13.2 ± 0.9%	T_g_—72 °C, T_max_—334 °C	-	586	33.7	[150]
13	Shape memory thermoplastic PU	MXene (Ti_3_AlC_2_)	-	T_g_—26.8 °C, T_max_—383 °C	-	341.1 W/g	-	[151]
14	Tris [2-(acryloyloxy)ethyl] isocyanurate	Diphenyl(2,4,6 trimethylbenzoyl)phosphine oxide	Tensile—48.7 MPa elongation—6% compressive strength—370.7 MPa	T_g_—280 °C, T_max_—454.4 °C	-	-	-	[152]
15	Poly(tetrahydrofuran) (PTMEG)	Phosphorus-containing diol (DPDF)	Tensile—41.8 MPa elongation—657.3% toughness—106.9 kJ/m^3^	T_g_—57.9 °C, T_max_—415 °C	24.8	1191.3	-	[153]
16	Furfuryl alcohol, polyethylene glycol, poly(propylene glycol)	Tri-maleimide end-capped cyclotriphospha zene	Tensile—19.8 MPa	T_max_—401.6 °C	23.9	362.8 W/g	8.74	[154]
17	DGEBA	(Aminooxy)diphenylphosphine oxide	Tensile—86.2 MPa elongation—11.6% toughness—6.8 MJ/m^3^	T_g_—118 °C, T_max_—390 °C	35.2	433.4	6.5	[155]
18	PTMEG, IPDI	DOPO	Tensile—54.5 MPa elongation—891.3% toughness—207.8 kJ/m^3^	T_g_—61 °C, T_max_—398 °C	-	744.3 W/g	-	[156]
19	Waterborne epoxy	Polydopamine-modified APP (PDA@APP)	-	T_max_—340.9 °C	32.6	278.8	38.7	[157]
20	Vanillin, DGEBA	Hexachlorocyclotriphosphazene	-	T_g_—82 °C, T_max_—253.3 °C	28.6	351.8	38.5	[158]
21	TA, PVA	Phosphoric acid	Tensile—5.4 MPa elongation—253% hardness (shore D)—38	T_max_—216 °C	75.7	-	-	[159]
22	DGEBA, eugenol	HCCP	Tensile—77.64 MPa elongation—4.4% impact strength—30.46 kJ/m^2^	T_g_—99.85 °C, T_max_—392.2 °C	26.2	346	16.4	[160]

T_max_—the temperature at which maximum degradation happens.
